# Electrical Identification and Selective Microstimulation of Neuronal Compartments Based on Features of Extracellular Action Potentials

**DOI:** 10.1038/srep31332

**Published:** 2016-08-11

**Authors:** Milos Radivojevic, David Jäckel, Michael Altermatt, Jan Müller, Vijay Viswam, Andreas Hierlemann, Douglas J. Bakkum

**Affiliations:** 1 ETH Zurich, Department of Biosystems Science and Engineering, 4058 Basel, Switzerland

## Abstract

A detailed, high-spatiotemporal-resolution characterization of neuronal responses to local electrical fields and the capability of precise extracellular microstimulation of selected neurons are pivotal for studying and manipulating neuronal activity and circuits in networks and for developing neural prosthetics. Here, we studied cultured neocortical neurons by using high-density microelectrode arrays and optical imaging, complemented by the patch-clamp technique, and with the aim to correlate morphological and electrical features of neuronal compartments with their responsiveness to extracellular stimulation. We developed strategies to electrically identify any neuron in the network, while subcellular spatial resolution recording of extracellular action potential (AP) traces enabled their assignment to the axon initial segment (AIS), axonal arbor and proximal somatodendritic compartments. Stimulation at the AIS required low voltages and provided immediate, selective and reliable neuronal activation, whereas stimulation at the soma required high voltages and produced delayed and unreliable responses. Subthreshold stimulation at the soma depolarized the somatic membrane potential without eliciting APs.

The structural and functional organization of neuronal circuits enables the brain to process sensory information, encode memories, think, and learn. It is generally acknowledged that these complex operations arise from orchestrated activities between individual neurons^1^. Therefore, the ability to simultaneously observe and elicit activity in selected individual neurons at cellular and subcellular resolution over extended periods of time will be pivotal for exploring basic governing principles in neural circuits^2^. Furthermore, the design of protocols for reliable and selective extracellular stimulation of individual neurons is an important task for developing neural prosthetics and brain-machine interfaces^3^, where advances in electrode-neuron interfacing technology and detailed characterization of neuronal responses to local electrical fields can lead to improved clinical outcomes.

A number of existing and emerging technologies aim to achieve this goal. For example, intracellular recording and regulation of neuronal activity can help decipher how information is regulated by synaptic plasticity or by the integration of subthreshold synaptic inputs^4^. While accurate readout and control of intracellular voltages are advantageous, the technique is limited to recording from, typically, a single or a few neuronal sites. Moreover, the duration of a recording session is limited to about an hour due to mechanical and biophysical instabilities from attaching a patch pipette to a cell. Recently designed fluorescent indicators or genetically encoded molecular probes offer a promising optical approach to record activity and precisely stimulate or inactivate individual neurons in a network^5^,^6^,^7^. A drawback is that these methods introduce foreign molecules inside the neurons, representing invasive and, at times, technically demanding approaches. Moreover, fluorescent indicators can interfere with cell physiology to the point of affecting action potential conduction^8^, and light exposure can lead to photobleaching of fluorescent indicators and cause phototoxic effects. Without introducing material inside the cell, infrared and near-infrared beams can activate or silence targeted neurons^9^,^10^. However, radiation energy is absorbed along its optical path and thereby reduces stimulation effectiveness, whereas increasing the beam power thermally damages exposed neurons^9^.

Microelectrode arrays (MEAs) are designed to extracellularly record and stimulate multiple neurons in a network. However, comprising low numbers of rather large electrodes, spaced at large pitch, standard MEAs do not allow for recording or selective stimulation of targeted neurons in the network. Emerging high-density microelectrode arrays (HD-MEAs) developed by other and our labs^11^,^12^,^13^,^14^,^15^ feature thousands of densely packed electrodes and give simultaneous access to large numbers of neurons at subcellular spatial resolution^16^,^17^,^18^. Owing to specific low-noise HD-MEA designs^13^,^15^, relatively small electrical signals arising from axons and proximal dendrites can be detected^18^,^19^.

Knowledge of optimal stimulation locations, effective stimulation magnitudes, and, ideally, of how to selectively stimulate any neuron in the network will benefit the design of extracellular electrophysiological tools and development of neural prosthetics^20^. However, conflicting reports exist in the literature about effective neuronal sites for electrical stimulation. Studies using single-unit extracellular electrodes in cortical brain slices suggest that axons but not cell bodies are activated by electrical stimulation^21^, and companion studies propose that axonal branches rather than the axon initial segment (AIS) react to extracellular charges^22^. In contrast, *in vivo* studies performed by using single glass extracellular electrodes in anesthetized cats reported that thresholds for activation of spinal motorneurons and spinal border cells were lowest near the AIS^23^. Studies performed on direction-selective ganglion cells of the rabbit retina showed that proximal axonal sections enriched in voltage-gated sodium channels have the lowest threshold for extracellular stimulation^24^. Comparable results were obtained with a HD-MEA system, suggesting that neuronal sites with the lowest extracellular stimulation threshold reside around 13 μm from the soma^25^. Capacitive extracellular stimulation directed along the axons of rabbit retinal ganglion cells revealed nearly constant thresholds except for a low-threshold region close to the cell soma^26^. Recent studies performed with HD-MEAs show that in contrast to axonal, dendritic extracellular stimulation is unlikely to provide effective activation of the neuron^19^. Modeling studies predict the AIS to feature sites with highest sensitivity to extracellular stimulation^27^,^28^. In the context of retinal prostheses, MEA systems were used to apply specific spatial patterns of current stimulation in an effort to target selected ganglion cells^29^. In addition, they were used to study complex responses of neocortical tissue induced by extracellular stimulation *in vivo*^30^.

In the present study, HD-MEAs, patch-clamp, and optical imaging of primary rat cortical cultures^13^ were combined in order to correlate morphological and electrical features of different neuronal compartments with their responsiveness to extracellular stimulation. Our objective was to provide a method for (i) noninvasive identification and (ii) selective stimulation of individual neurons in large cortical networks interfaced with complementary-metal-oxide-semiconductor (CMOS) -based HD-MEAs. Herein we demonstrate strategies to electrically identify neuronal compartments of any neuron in the network, purely based on extracellular features of their APs. We propose analytical approaches to reveal correlations between spatiotemporal features of extracellular APs and effective stimulation voltages, and we explore the limits of targeted extracellular stimulation of different neuronal compartments (soma, AIS, proximal and distal axonal segments). Finally, we demonstrate a method to achieve selective orthodromic and antidromic stimulation while using low stimulation voltages, and show an approach to modulate subthreshold membrane potentials through somatic extracellular stimulation.

## Results

### Identification of individual neurons and their axonal arbors in a network

**Figure 1:**
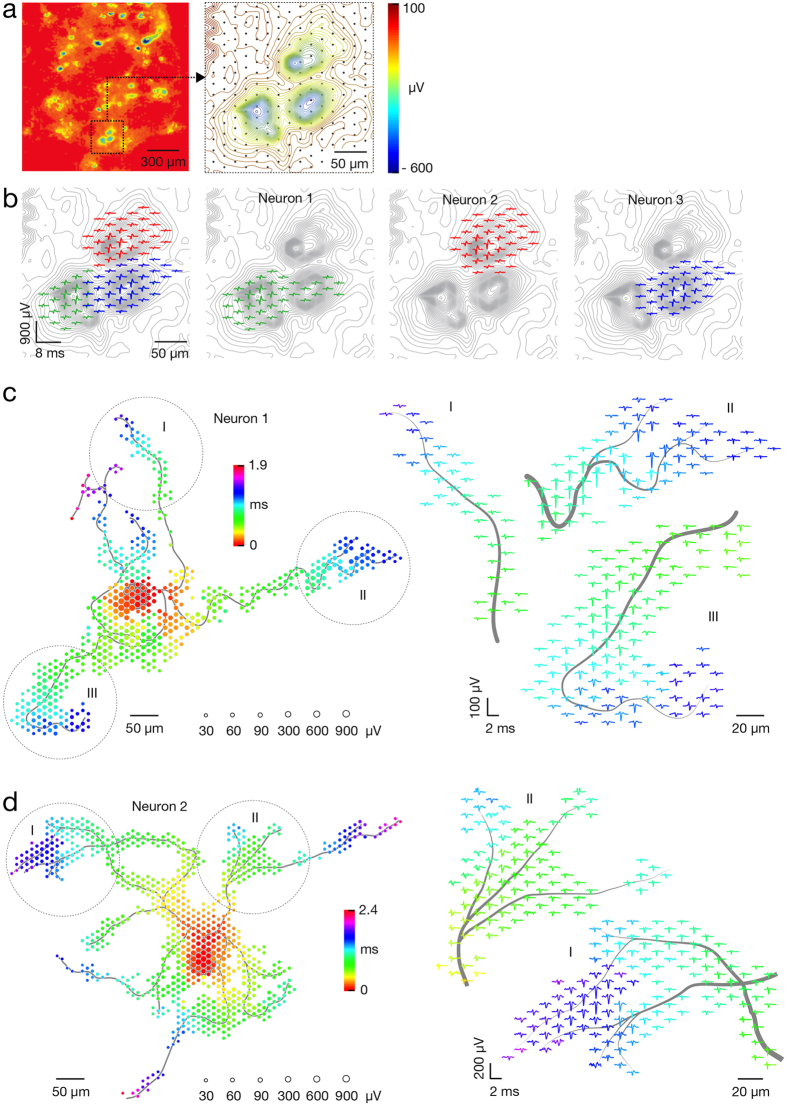
Electrical identification of individual neurons on the array based on their spontaneous activity. (**a**) Spontaneous activity map of cortical neuronal culture. Amplitudes of 60–300 APs recorded by each electrode were averaged and color-coded. The selected region of the activity map has been magnified 6 times; averaged signals have been interpolated and are represented through equipotential lines; black dots in the background represent the electrode locations. (**b**) Footprints of spontaneous AP activity of three neurons (colored in green, red and blue) in the selected region of the activity map in (**a**). Superimposed (left) and individual electrical activity (labels ‘Neuron 1–3’). (**c,d**) Spike-triggered-average footprints of ‘Neuron 1’ and ‘Neuron 2’ are presented at the left of (**c**,**d**). Circle sizes indicate logarithmically scaled amplitudes of APs, whereas colors indicate occurrence times of negative AP peaks relative to first AP activity of the respective neuron. Three selected regions of each spike-triggered average footprint (labeled I, II and III) are magnified 2.5 times and presented at the right of (**c**,**d**). Gray axonal contours serve as guide to the eye and are estimated by observing the spatial movement of signal peaks in consecutive movie frames. The footprints of ‘Neuron 1’ and ‘Neuron 2’ are also presented in Supplementary Movie S1.

We utilized the bidirectional interface of our HD-MEA to develop a set of recording and stimulation strategies for identifying individual neurons and their axonal arbors. To initially identify the location of neurons, a sequence of recording configurations was scanned across the array while recording spontaneous neuronal activity for a couple of minutes per configuration. Figure 1a presents a resulting activity map. Since the largest extracellular signals typically occur in regions proximal to the soma^18^,^19^, local minima (regions of largest negative action potential values) in the activity maps can be used to indicate the approximate neuronal locations. In many instances, however, electrical identification of a single neuron was difficult because multiple somas resided close to each other and their signals overlapped. However, ‘spike sorting’ of neuronal activity, recorded with high-spatial-resolution electrode arrays, allows identifying individual neurons^17^,^31^,^32^. After spike sorting, we reconstructed a neuron’s spatially distributed extracellular field, termed a spontaneous AP footprint, by averaging 40–60 AP waveforms. Figure 1b shows footprints of 3 adjacent neurons. The spatiotemporal distribution of AP waveforms with respect to neuronal morphology is presented in Supplementary Fig. S3. In general, spontaneous AP footprints include large extracellular signals in the regions of the AIS and other proximal neuronal compartments, while much smaller signals are observed in the regions of the proximal dendrites and axons.

After identifying a target neuron, a higher resolution footprint was acquired by scanning recording configurations across the array, as before, but now also always recording spikes from the target neuron. Spike-triggered averaging, triggered by the target neuron, now revealed signals arising from its axonal arbor^18^,^19^. Spiking activity was recorded for 2 minutes per configuration and, again, averaged over a minimum of 40–60 waveforms, with this averaged signal being termed a spike-triggered average footprint (Fig. 1c,d and Supplementary Movie S1,2,3–4). Owing to the favorable signal-to-noise characteristics of the HD-MEA (2.4 μV_RMS_ in the AP band), it was sufficient to average 40–60 AP waveforms in order to reliably reconstruct the AP footprints across axonal arbors^18^,^19^ (see also Supplementary Fig. S1a–d). In general, spike-triggered averaging reliably identified tonically active neurons in the network. Network bursts, when many axons are simultaneously active, degraded reconstructions of AP waveforms if the same recording sites captured signals from different neurons simultaneously. To avoid this scenario, we detected network bursts^33^ and excluded APs in bursting periods.

**Figure 2:**
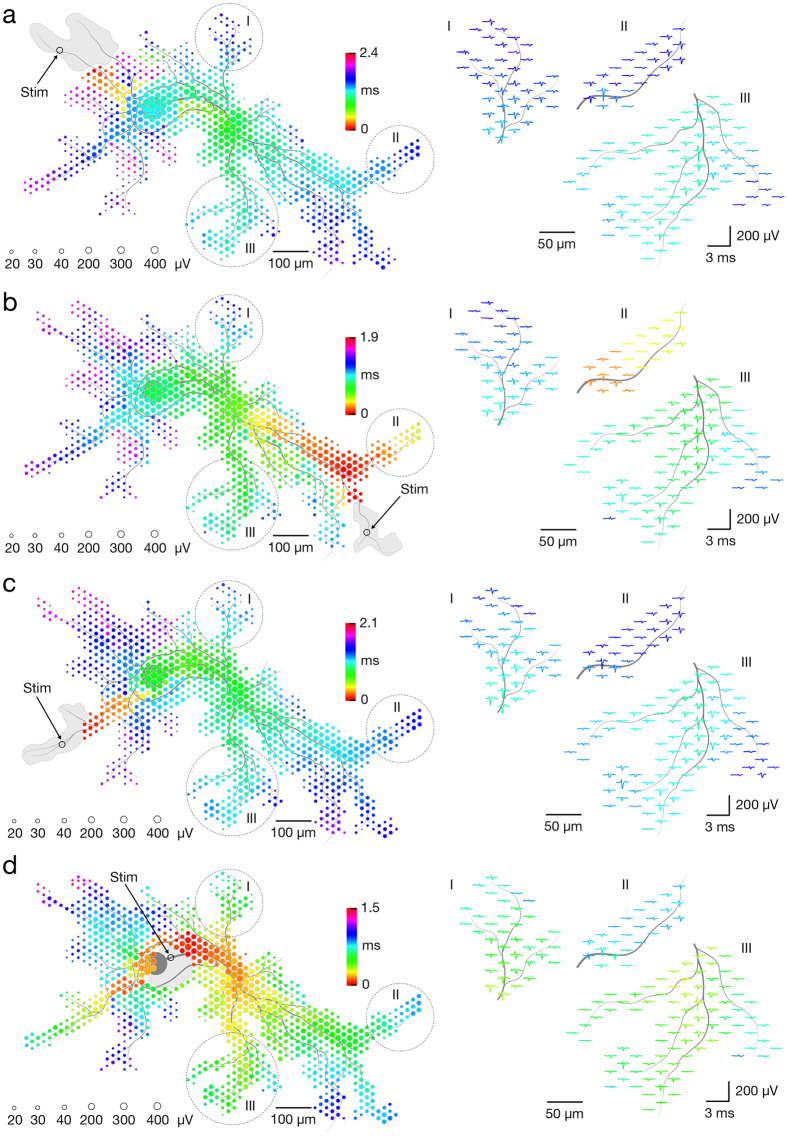
Electrical identification of individual neurons on the array based on their responses to extracellular stimulation. Footprints of a single neurons as obtained from consecutively triggering 4 different stimulation sites are presented in panels (**a**–**d**). Circle sizes indicate logarithmically scaled average amplitudes of evoked APs, whereas colors indicate occurrence times of AP’s negative peaks relative to the stimulation time. Black hollow circles indicate stimulation sites, and gray patches delineate areas affected by the stimulation artifact. Selected regions of stimulation-triggered footprints (labeled I, II and III) are magnified 2 times and presented in the right part of each panel. Gray axonal contours serve as guide to the eye and are estimated by observing the spatial movement of signal peaks in consecutive movie frames. The four stimulation-triggered footprints are also presented in Supplementary Movie S2. Waveforms of averaged and individual APs at 3 distal axonal compartments are given in Supplementary Fig. S1.

Some ‘silent’ neurons in the network had infrequent or no activity^34^, such that averaging spontaneous spikes did not produce clear footprints. However, such neurons could be activated by voltage-controlled extracellular stimulation. Their footprints were, therefore, acquired by scanning recording configurations across the array, as before, while now stimulating a given electrode multiple times per configuration, termed a stimulation-triggered footprint (Fig. 2a–d and presented in Supplementary Movie S2). We used charge-balanced positive-first biphasic voltage pulses with phase durations of 200 μs and amplitudes between ±10 and ±1,000 mV, delivering charges between approximately 16 and 1,600 pC (see Methods section for further details). Stimulation could evoke multiple neurons. Therefore, we identified individual neurons by systematically applying orthodromic and antidromic stimulation at different locations and comparing the responses. Stimulation voltages were gradually decreased until the neuron of interest was selectively stimulated at multiple sites. For a comparison, waveforms of averaged and individual APs at 3 distal axonal compartments are given in Supplementary Fig. S1.

In addition to identifying silent neurons in the network, stimulation-triggered recordings could cleanly identify footprints of neurons active during network bursts. In such cases, spike- and stimulation-triggered footprints were compared to confirm the topographical match of detected axonal arbors. Spontaneous and stimulation-triggered activities of the same neuron are presented in Supplementary Movie S3. In general, spike- and/or stimulation-triggered averaging of neuronal signals enabled us to identify neurons regardless of their activity level.

**Figure 3:**
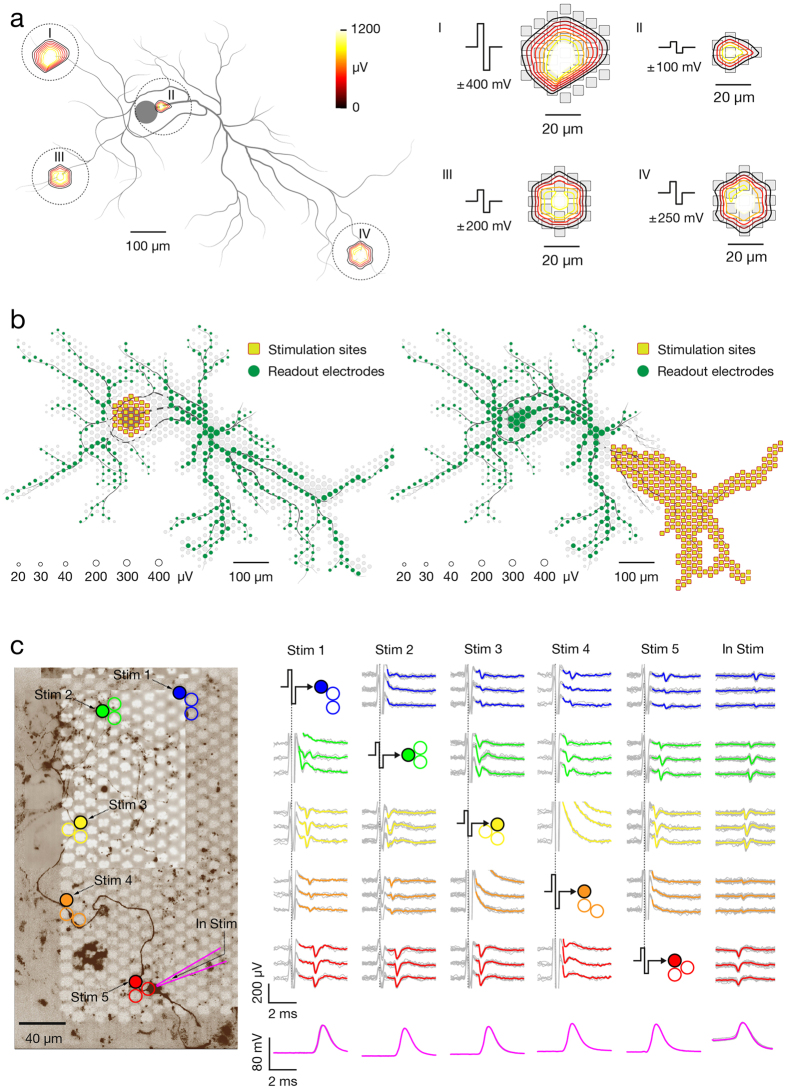
Bidirectional electrode-neuron communication enables studying responses of different neuronal compartments to extracellular stimulation at subcellular spatial resolution. (**a**) (Left) Artifacts caused by extracellular stimulation at four different neuronal sites labeled I, II, III and IV. Average amplitudes of artifact signals recorded by the respective electrodes are color-coded, interpolated, and presented as equipotential lines. (Right) Artifacts are superimposed to corresponding electrodes (displayed in gray). The stimulation voltage pulses are also displayed. Gray axonal contours serve as guide to the eye and are estimated by observing the spatial movement of signal peaks in consecutive movie frames. (**b**) Examples of electrode configurations used for studying responses to extracellular stimulation of somatodendritic (Left) and axonal (Right) compartments. Green circles represent recording electrodes, and their sizes indicate logarithmically scaled average amplitudes of the recorded APs. Yellow squares represent stimulation electrodes. Gray circles in the background represent the neuron’s spike-triggered average footprint. Gray axonal contours serve as guide to the eye and are estimated by observing the spatial movement of signal peaks in consecutive movie frames. (**c**) (Left) Fluorescence image (Fluo4 calcium dye, background) indicating the neuronal morphology. MEA electrodes can be seen as white squares, whereas five groups of three electrodes, represented by colored circles, were used for experiments. Electrodes denoted by filled circles were also utilized for stimulation. The cell was patched at its soma (illustrated by the purple pipette) in current clamp mode. (Right) First five columns (denoted as ‘Stim 1–5’) represent experiments, where extracellular stimulation was applied through one of the 5 stimulation electrodes, while neuronal signals were recorded extracellularly and intracellularly. The last row (denoted as ‘In Stim’) represents an experiment in which the neuron was stimulated intracellularly. Stimulation pulses and averaged extracellular APs are color-coded according to locations indicated in the fluorescence image. Averaged intracellular traces are colored in purple. Raw extracellular and intracellular signals are displayed in gray.

Stimulation artifacts obscure neuronal responses close to the stimulation site, where the extracellular AP is largest, so that determining whether or not a stimulus evoked a nearby neuron is difficult. Some methodologies have been developed to reduce artifacts^35^,^36^. Advantageously, HD-MEAs can detect small signals from neuronal processes distant to the stimulation site that are not affected by stimulation artifacts (Fig. 3a,b). Stimulation-triggered averaging of axonal signals then enabled us to reliably measure the efficacy of extracellular stimulation applied to any site of the neuron. While stimulation artifacts affected many electrodes transiently, only artifacts on electrodes within a 10 to 60-μm radius of the stimulation site obscured footprints. In general, the artifact area depended on stimulation magnitude (Fig. 3aI–IV). Artifacts from higher voltages (±400–±1000 mV) saturated ~60–100 electrodes (Fig. 3aI); lower voltages (±30–±100 mV) saturated ~7 electrodes within a ~20-μm radius (Fig. 3aII). To ensure reliable detection of stimulation-triggered APs arising from different neuronal compartments, including complex axonal arbors, we created configurations to match neuron ‘functional’ morphology by selecting recording electrodes whose averaged signals exhibited a signal-to-noise ratio (SNR) of at least 4 standard deviations. Such selection of recording electrodes enabled us to reliably detect individual APs across multiple axonal compartments (Supplementary Fig. S1), with captured signals reflecting a neuron’s ‘functional’ morphology (Fig. 3b). In many instances, particularly when higher stimulation voltages were applied, multiple neurons in the network were simultaneously evoked, and some recording electrodes captured mixed signals from different compartments of multiple neurons. Owing to custom-designed recording configurations based on ‘functional’ morphology, individual neurons could still be discerned by analyzing signals at electrodes that did not capture mixed signals. Scenarios where two neurons have similar morphology, occupy the same recording electrodes, and have similar waveforms were considered unlikely.

To verify our approach, we combined HD-MEA and whole-cell patch-clamp recordings/stimulations of a single neuron. We stimulated proximal, middle and distal regions of the axon while recording extracellular axonal signals and intracellular somatic APs (Fig. 3c). This procedure enabled (i) optical observation of the neuron of interest in order to verify the origin of extracellular APs, (ii) artifact-free intracellular recordings to indicate if extracellular stimulation was successful, and (iii) intracellular stimulation to verify how well extracellular recordings can detect APs. Figure 3c shows an individual neuron and the location of five clusters of recording electrodes (3 electrodes per cluster) under parts of its axon. One electrode per cluster was also used for stimulation. Action potentials, elicited by stimulation at each of the five neuronal sites, were simultaneously detected with the patch-clamp electrode and extracellular electrodes. Detection of intracellular and extracellular signals coincided in 100% of trials (40 stimuli were applied per site). We observed consistent results from 4 different neurons in 4 different cultures. It is also evident from Fig. 3c that elicited electrical activity always spreads in both directions, orthodromically and antidromically, along the axon from the stimulation site.

### The site with the lowest threshold for extracellular stimulation is near the peak of the electrical footprint

**Figure 4:**
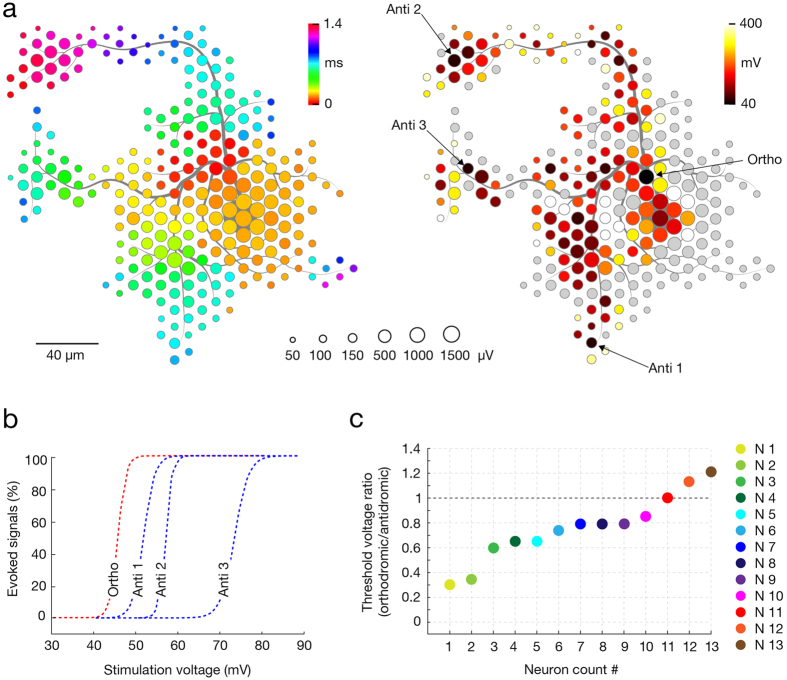
The core of neuron’s spontaneous AP footprint comprises site with lowest threshold for extracellular stimulation. (**a**) (Left) Spike-triggered average footprint of a single neuron. Circle sizes indicate logarithmically scaled amplitudes of APs, whereas colors indicate occurrence times of AP’s negative peaks relative to first AP activity of the respective neuron. (Right) Stimulation map covering the neuron’s spike-triggered average footprint. Site-specific stimulation thresholds are color-coded, whereas sites that did not provide neuronal activation when stimulated with ±600 mV (corresponding to ~942 pC) are colored in gray. The four sites with the lowest stimulation thresholds include three antidromic stimulation sites, labeled ‘Anti 1–3’, and one orthodromic stimulation site, labeled ‘Ortho’. Gray axonal contours serve as guide to the eye and are estimated by observing the spatial movement of signal peaks in consecutive movie frames. (**b**) Excitability profiles of the four sites with the lowest stimulation thresholds. Profiles of the orthodromic and antidromic stimulation sites are colored in red and blue, respectively. (**c**) Stimulation thresholds for the most sensitive orthodromic and antidromic sites were determined for 13 neurons. The ratio of lowest thresholds at ortho- and antidromic stimulation sites were calculated for each neuron, ordered in magnitude and are displayed. Different colors refer to different tested neurons, labeled as ‘N1–13’.

Neuron-wide stimulation over a range of voltages revealed the spatial distribution of sites with low thresholds for extracellular stimulation. The stimulation site featuring the lowest threshold typically was found within the center region of the neuron’s spike-triggered average footprint (Fig. 4). The stimulation threshold was defined as the minimal voltage that triggered an AP on the readout electrodes in 100% of the trials. Stimulation was applied at 4 Hz for voltages from ±10 to ±1,000 mV, with steps of ±10 mV. The stimulation frequency of 4Hz was selected as it is, on the one hand, not high enough to affect the reliability of neuronal responses, but, on the other hand, sufficiently high to allow for consecutive stimulation of hundreds of electrodes within 2 hours. Each stimulation voltage was applied 60 times per site. Since stimulation could possibly affect intrinsic neuronal plasticity^37^, stimulation sites and voltage magnitudes were randomized throughout experiments. Stimulation protocols were either limited to 2 hours or, if longer, were interrupted with ~30 minutes breaks. Figure 4a represents a ‘stimulation map’ (right) applied at locations of the neuron’s spike-triggered average footprint (left), revealing four stimulation sites (3 antidromic and 1 orthodromic) with the lowest thresholds. To get more precise excitability profiles, the most sensitive sites were then stimulated with voltages stepped by 1mV (60 stimuli per step in random order) (Fig. 4b). We produced stimulation maps for 13 neurons in total and extracted stimulation threshold for the most sensitive orthodromic and antidromic stimulation sites. The ratios between lowest stimulation thresholds at orthodromic and antidromic stimulation sites (orthodromic/antidromic) were calculated for each neuron, ordered in magnitude and are displayed in Fig. 4c. In 10 out of 13 neurons tested, an orthodromic site was the most effective stimulation site (Neurons 1–10), in one case, stimulation threshold values were identical (Neuron 11), and in two cases, an antidromic site had the lowest threshold (Neurons 12 and 13).

### Stimulation of the most sensitive site, found in the region of AP initiation, provides selective activation of an individual neuron

**Figure 5:**
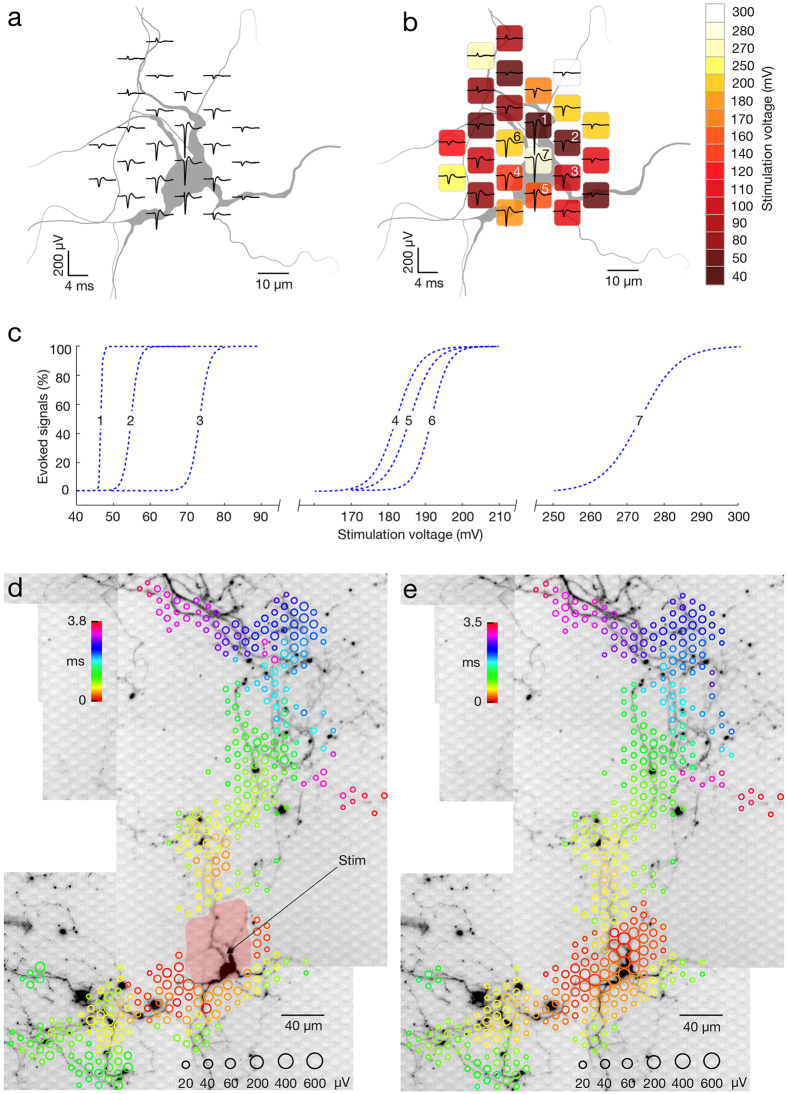
Selective stimulation of single neurons. (**a**) Spontaneous activity footprint (black traces) superimposed over the morphology of the respective neuron and its proximal compartments (colored in gray). Neuronal morphology was inferred from a microscopy image of the lipofected neuron. (**b**) Stimulation map superimposed over the footprint and neuron morphology (same neuron as in A). Site-specific stimulation thresholds are color-coded, and the electrodes in the center of the spontaneous activity footprint are numbered (1–7). (**c**) Excitability profiles for stimulation through the 7 center electrodes. Numbers correspond to those in (**b**). (**d**) Stimulation-triggered footprint superimposed over a micrograph of the corresponding lipofected neuron. Circle sizes indicate logarithmically scaled amplitude of triggered APs, whereas colors indicate the occurrence times of negative AP peaks relative to the stimulation time. The black arrow points to the stimulation electrode for orthodromic stimulation, whereas the pale red patch indicates the area affected by the stimulation artifact. The neuron was selectively stimulated 60 times at 4 Hz at the stimulation threshold value (±50 mV, corresponding to ~31 pC). These data are also presented in Supplementary Movie S5. (**e**) Spike-triggered average footprint of spontaneous electrical activity superimposed over the micrograph of the same lipofected neuron as in (**d**).

Focusing on proximal neuronal compartments, we found that stimulation of the most sensitive site provided selective single-cell neuronal activation in 96.2% of the cases (50 out of 52 neurons) when voltages at threshold values were applied (selective in this context means that only the target neurons were activated). We performed stimulation-triggered recordings to construct stimulation maps (Fig. 5b) and excitability profiles (Fig. 5c) for 52 different neurons in 27 preparations. For all neurons, 1–3 sites had steep excitability profiles with remarkably low activation thresholds (example in Fig. 5c, electrodes 1, 2, 3), and the most sensitive sites typically resided near the peak of the AP footprints (example in Fig. 5b, electrode 1). In general, activation thresholds of the most sensitive sites had values between ~±40 and ~±90 mV, whereas two extreme cases rendered values of ±20 and ±30 mV. To further verify whether threshold stimulation at the most sensitive site provided selective activation of a single neuron, or it also triggered other neurons in the culture, we compared stimulation-triggered footprints (see example in Fig. 5d) with spontaneous spike-triggered footprints (see example in Fig. 5e). As low voltages were needed to stimulate the most sensitive sites, stimulation artifacts were small (example in Fig. 3aII) such that signals beyond ~20-μm radius of the stimulation site were detectable (Fig. 5d). However, as stimulation artifacts often distort the AP waveforms that can be detected on neighboring electrodes, we used a larger safety margin in order to avoid any artifacts that would compromise the comparison between spontaneous and elicited activity in Fig. 5d. Lipofection provided sparse labeling of cultured neurons and was done to verify that ‘functional’ morphology matched neuron morphology (Fig. 5a,d,e). The response of a lipofected neuron to selective stimulation is presented in Supplementary Movie S5. Nonselective stimulation of other neurons in the culture was evident by detecting their orthodromic and antidromic axonal signals, followed by a clearly noticeable largest spike within the respective neuronal footprint. Comparison between selective and nonselective stimulations is presented in Supplementary Movie S4.

### Correlation of footprint features and stimulation threshold

**Figure 6:**
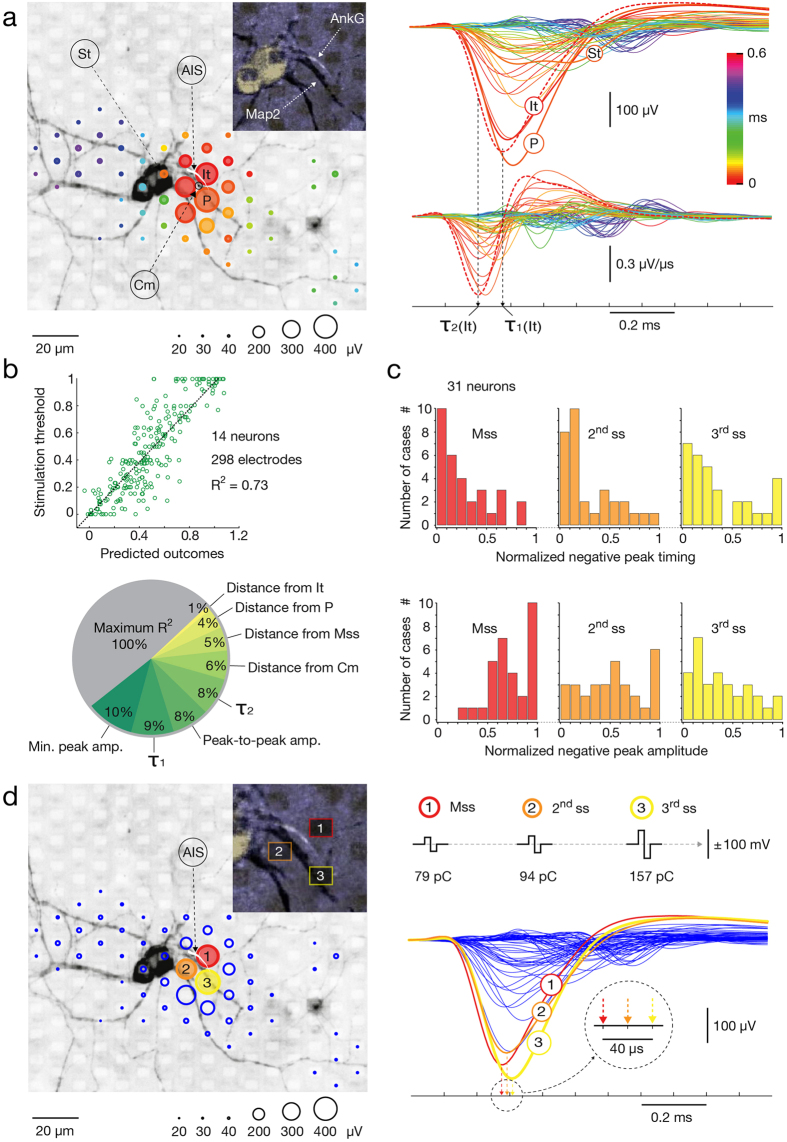
Correlations between spontaneous AP footprints, stimulation thresholds, and morphology of proximal compartments. (**a**) (Left) Spontaneous footprint superimposed over a fluorescence image showing the corresponding neuronal morphology (gray; Map2) and the AIS (white; AnkG). Magnified inset focusing on the AIS is presented in left upper angle of the panel. Circle sizes: amplitudes of APs; color: occurrence time relative to the first AP, denoted as It. The largest amplitude (P), nearest the soma (St), and the ‘center of mass’ (Cm) traces of the footprint are labeled. (Right) Superimposed AP traces (top) and their 1^st^ derivatives (bottom). Color: occurrence time relative to It; dashed lines: the initial trace and its 1^st^ time derivative; black arrows pointing to τ_1_(It) and τ_2_(It) indicate their negative peak values. (**b**) (Top) Least squares regression analysis of parameters extracted from stimulation maps and corresponding footprints of 14 neurons. The regressors were: (1–4) spatial distance of the stimulation site from It, P, Cm and the most sensitive site (Mss); (5) occurrence time of the negative peak (τ_1_); (6) occurrence time of the minimum in the 1^st^ derivative (τ_2_); (7) peak-to-peak amplitude and (8) negative peak amplitude. (Bottom) Pie diagram of the relative reduction (%) of the R^2^ value by removing individual regressors from the overall parameter space (green palette). R^2^ values obtained with all 8 regressors are in gray. (**c**) Normalized AP amplitude and timing distributions at the three most sensitive sites (Mss) of 31 neurons. Distributions of most, second and third sensitive sites (Mss, 2^nd^ss, 3^rd^ss) are presented in red, orange and yellow histograms. (**d**) (Left) Positions of the 3 most effective stimulation sites and location of the AIS (same neuron as in (**a**)). Most, second and third sensitive sites are marked with red, orange and yellow circles. (Right) Extracellular AP traces, recorded near the 3 most sensitive sites, are highlighted in the full set of traces. Stimulation threshold voltages (50, 60, 100 mV) and corresponding charges (~79, ~94, ~157 pC) for the 3 most sensitive sites are at the top. The occurrence times of the negative peaks at these 3 electrodes are in the inset.

To investigate if sensitivities of sites in proximal neuronal compartments can be predicted by features of the extracellular AP footprint, we correlated stimulation thresholds with various parameters of the amplitude, spatial and temporal distribution of extracellular voltage traces. While individual parameters showed no correlations with stimulation thresholds (Supplementary Fig. S2a,b), a set of parameters, obtained by least squares regression analysis, revealed statistically significant correlation with stimulation thresholds (Fig. 6b).

We proceeded by reconstructing AP footprints of 14 neurons and finding their stimulation maps. Voltage traces with peak amplitudes of <20% of the footprint peak were excluded. The parameters extracted from the footprints for each electrode were: (1) peak-to-peak amplitude, (2) negative peak amplitude, (3) time of the minimum peak, (4) time of the maximum falling slope (i.e. peak of the first derivative) (Fig. 6a). These parameters were correlated with the corresponding stimulation thresholds but only revealed statistically negligible R^2^ values (Supplementary Fig. S2a). Further distance parameters were calculated between the location of the stimulation site and the location of: (1) the trace with the largest negative amplitude (P), (2) the first emerging AP signal within the footprint (It), (3) the neuronal site that revealed lowest stimulation threshold (Mss) and (4) the footprint’s center-of-mass (Cm) (Fig. 6a). The center-of-mass coordinates were calculated as follows: *X*_*CM*_ = *∑*_*i*_ *Amp*_*i*_ *x*_*i*_*/∑*_*i*_ *Amp*_*i*_; *Y*_*CM*_ = *∑*_*i*_ *Amp*_*i*_ *y*_*i*_*/∑*_*i*_ *Amp*_*i*_; where *Amp*_*i*_ represents the negative peak amplitude captured on electrode *i* with *x*_i_ and *y*_*i*_ coordinates on the array. Again, these parameters were correlated with the corresponding stimulation thresholds, but we did not find statistically significant R^2^ values (Supplementary Fig. S2b).

We next tested if a least squares regression analysis of the above parameters could predict site-specific stimulation thresholds within the area of the spontaneous AP footprint. To test our hypothesis, the eight parameters were utilized as regressors and stacked within the design matrix, whereas the site-specific stimulation thresholds were treated as a measured variable. We correlated site-specific stimulation thresholds with the scalar product of the design matrix and the corresponding regression coefficients, and found statistically significant correlation, rendering low p value (p < 0.001) and an R^2^ value of 0.73 (Fig. 6b). The possibility to make predictions with the obtained model was tested on 17 different, newly obtained datasets, but the performance in predicting the 3 most excitable sites for extracellular stimulation was comparably poor. We next sequentially eliminated individual parameters from the design matrix and calculated how the R^2^ value decreased. While sequential elimination of individual parameters about the timing and amplitude of the negative traces in the footprint decreased the initial R^2^ value for 8–10%, removal of parameters about positional information of the stimulation site had weaker influences on the R^2^ value (Fig. 6b).

Guided by this observation, we investigated if precise locations of the three most excitable sites (most sensitive, second and third sensitive sites, Mss, 2^nd^ss and 3^rd^ss) residing near the largest and first recorded traces of the footprint can be associated with amplitudes and timing of the extracellular AP waveforms (Fig. 6c). We constructed spontaneous AP footprints of 31 neurons and found their stimulation maps. Amplitude and timing of the footprints’ negative signal peaks were extracted and ranked. Distributions of the three most excitable sites were shifted towards the largest and earliest traces in the neuron’s footprint with regard to amplitudes and timing of extracellular APs (Fig. 6c). In 80.6% of cases, the most excitable sites co-localized with the first recorded trace of the footprint (10 out of 31 cases for Mss, 8 out of 31 cases for 2^nd^ss and 7 out of 31 cases for 3^rd^ss), whereas in 58.1% of cases, the most excitable sites co-localized with the trace with the largest negative amplitude (10 out of 31 cases for Mss, 6 out of 31 cases for 2^nd^ss and 2 out of 31 cases for 3^rd^ss).

In order to optically correlate the position of the AIS with regard to locations of the most sensitive sites and the footprint, cultures were immunostained against microtubule-associated protein-2 (Map2) and ankyrin-G (AnkG) to determine the morphology of the somatodendritic compartments and AIS (Fig. 6a,d and Supplementary Fig. S2a). For this purpose, we seeded low-density neuronal cultures (~1000 cells per array) that provided sparse spatial distribution of individual neurons in the cultures and facilitated their electrical and visual identification. Because the first recorded and largest trace of the footprint co-localize with distal and central-to-proximal regions of the AIS, respectively (D. Bakkum, 2016, manuscript submitted; please see discussion), we used these AIS indicators to quantify distances of the three most effective electrodes. Again, the three electrodes that required the lowest voltages to effectively stimulate the cell were found to be in closest proximity to the AIS (Fig. 6d). Consistent results were obtained from three cells in total, in each of which, the most effective electrode co-localized with the first-emerging trace of the footprint. Distances from the first recorded trace were 21 ± 16 μm for Mss, 25 ± 22 μm for 2^nd^ss and 34 ± 24 μm for 3^rd^ss (mean ± std). Distances from largest trace were 16 ± 12 μm for Mss, 31 ± 18 μm for 2^nd^ss and 37 ± 21 μm for 3^rd^ss (mean ± std)). The locations of the AIS landmarks have been determined in reference to the positions of electrodes that recorded the first and largest-amplitude electrical activities within the respective neuronal footprint. The distance values, therefore, refer to inter-electrode pitches between stimulation electrodes and the respective recording electrodes.

### Stimulation applied at the AIS provides immediate elicitation of APs, whereas somatic stimulation induces slow subthreshold deflections in the neuron’s membrane potential

**Figure 7:**
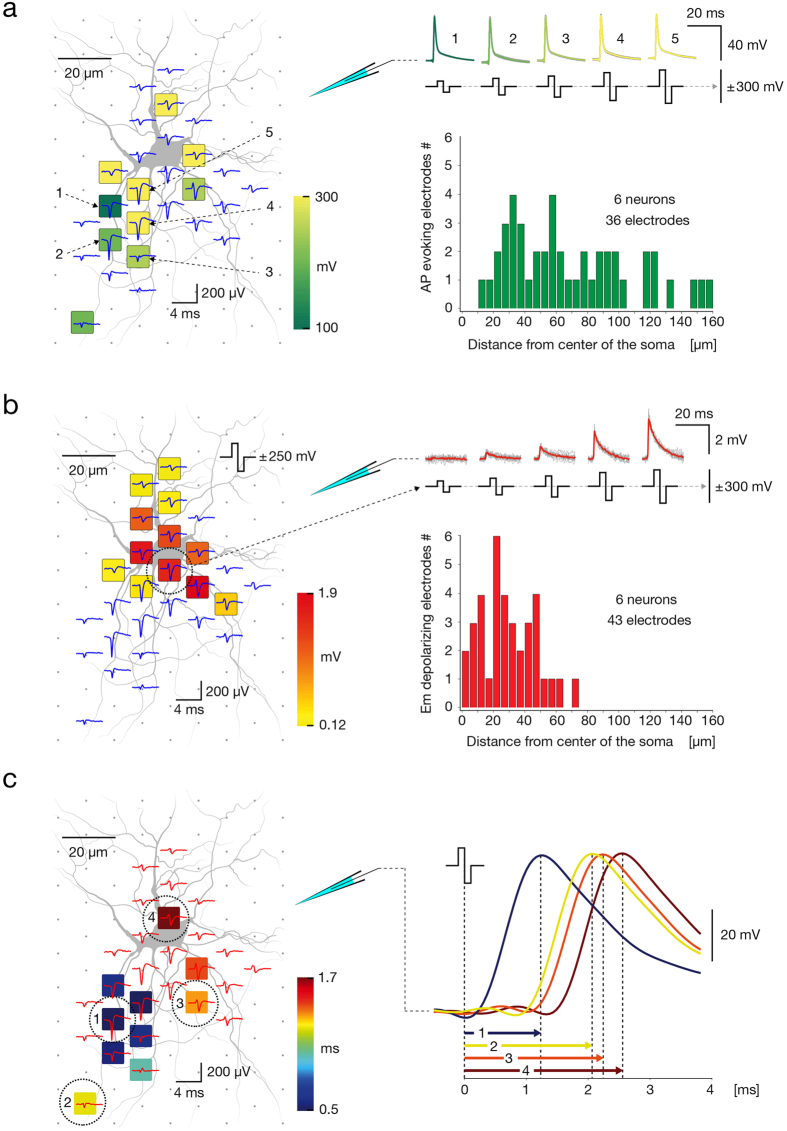
Responsiveness to supra- and subthreshold extracellular stimulation of proximal neuronal compartments. (**a**) (Left) Stimulation map superimposed over the spontaneous AP footprint (blue traces) and the morphology of proximal neuronal compartments, obtained through staining. Site-specific stimulation thresholds are color-coded, and five electrodes closest to the putative AIS are numbered (1–5). (Right top) Averaged intracellular APs, measured through patch clamp, that have been triggered by threshold stimulations (depicted as biphasic pulses) applied through the five numbered electrodes are color-coded and superimposed over individual signals (gray). (Right bottom) Distribution of the distances between electrodes that evoked APs and the soma center. For all cells, stimulation voltages up to ±300 mV (~471 pC) were used. (**b**) (Left) Subthreshold-stimulation map superimposed over the spontaneous AP footprint (blue traces) and morphology of proximal neuronal compartments obtained through staining. Magnitudes of stimulation-triggered subthreshold signals (measured by patch clamp) are color-coded. One specific electrode near the soma, marked with a dashed circle, was used for extracellular stimulation. (Right-top) Intracellular patch clamp signals evoked by subthreshold stimulations (depicted as biphasic pulses) through the electrode near the soma. Signal averages are color-coded; individual signals are displayed colored in gray. (Right bottom) Distribution of the distances between stimulation electrodes inducing membrane potential depolarizations and the respective soma center. For all cells, stimulation voltages up to ±300 mV (~471 pC) were used. (**c**) Activation-time map superimposed over the spontaneous AP footprint (red traces) and morphology of the proximal neuronal compartments. Lengths of activation intervals (time between extracellular stimulation at the respective electrode and occurrence of the intracellular AP at the patch electrode) are color-coded. Four representative stimulation electrodes are numbered (1–4), and the corresponding evoked intracellular signals are displayed at the right. Neuronal morphology in (**a**–**c**) was obtained through fluorescence labeling by using a Fluo4 calcium dye.

We found that while suprathreshold extracellular stimulations applied near locations of the AP’s largest extracellular traces evoked APs (Fig. 7a), subthreshold stimulations applied at the same sites caused no detectable effect on the somatic membrane potential, even when voltages with 95% of the corresponding threshold values were applied. In contrast, extracellular stimulation applied near the soma induced subthreshold deflections in the neuron’s membrane potential and only gave rise to APs if relatively high stimulation voltages were applied (Fig. 7b). Individual neurons on the array were fluorescently labeled by injecting intracellular dye through the patch pipette. Bath application of synaptic blockers abolished synaptic inputs and consequently reduced spontaneous activity. Therefore, AP footprints were constructed mainly based of averaging of extracellular responses triggered from the patch-clamp electrode, and rarely based on spontaneous activity. Proximal neuronal compartments were extracellularly stimulated across voltage steps of ±1mV, and patch-clamp recorded signals were classified into a sub- and suprathreshold responses. The stimulation map presented in Fig. 7a depicts stimulation sites and corresponding minimum voltages required to effectively trigger an AP with 100% fidelity. The positions of effective stimulation sites obtained from 6 neurons (36 electrodes in total) were compared to the centers of their somas (see histogram in Fig. 7a). The map presented in Fig. 7b, referred to as a subthreshold map, shows positions of electrodes and magnitudes of changes in the neuron’s membrane potential induced by subthreshold stimulation from these electrodes. We constructed subthreshold maps for 6 neurons (43 electrodes in total), and the positions of electrodes inducing changes in subthreshold membrane potential were compared to their somatic centers (see histogram in Fig. 7b). The ability to directly induce subthreshold depolarization was typically constrained to electrodes located in close proximity to somatic regions, and the depolarization magnitudes were proportional to the applied stimulation voltages (see example in Fig. 7b). In general, these electrodes could evoke an AP when high stimulation voltages were applied (>±400 mV). In some of cases, however, suprathreshold stimulation was not possible within a safe range of voltage magnitudes (i.e. within ±1000 mV).

We next utilized patch-clamp recordings to estimate the time delay between extracellular stimulation and the somatic intracellular AP. We found that extracellular stimulations applied near locations of the AP’s largest extracellular traces provided the fastest intracellular responses, while stimulation of sites that conveyed smaller extracellular traces led to later appearing APs (Fig. 7c). For these results, we constructed AP footprints and produced corresponding stimulation maps for 4 neurons. Delay was calculated between the time of the stimulation pulse downswing and the occurrence of the responding intracellular AP’s peak (Fig. 7c right). Activation-time maps were produced by assigning activation delays to corresponding stimulation electrodes (Fig. 7c left).

### The electrode with the initial AP trace has the most reliable response to a high-frequency extracellular stimulation

**Figure 8:**
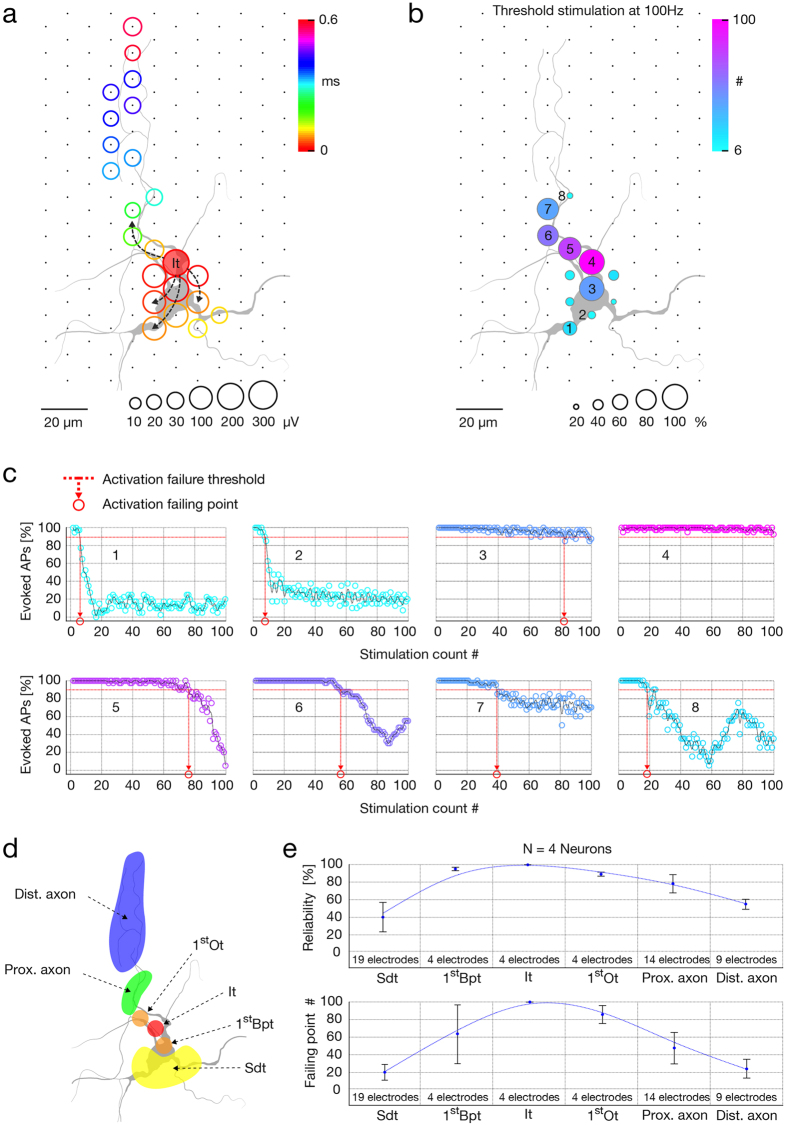
Reliability of neuronal response for high-frequency extracellular stimulation. (**a**) Spike-triggered average footprint superimposed over the neuronal morphology as obtained by lipofection. Circle sizes represent logarithmically scaled amplitudes of spontaneous APs, whereas colors indicate occurrence times of the AP negative peaks relative to the first recorded activity (It). The initial trace of the spike-triggered average footprint is marked as It, while the spreading of the extracellular AP is indicated with dashed arrows. (**b**) Activation reliability map. Circle sizes indicate relative activation reliability (%), and the colors represent activation failing points (APs not evoked, see (**c**) below). Activation reliability represents the percentage of successfully evoked responses, whereas activation failing points represent the number of stimulations, after which activation reliability dropped below 95%. Eight electrodes (numbered as 1–8) were used to stimulate at 100 Hz with the respective activation threshold voltage. (**c**) Reliability profiles for the eight electrodes indicated in (**b**). Color corresponds to that in (**b**), stimulation frequency was 100 Hz. Red dashed lines indicate activation failing thresholds and red circles projected on the x-axes indicate the activation failing points. The analysis has been repeated with fixed, high stimulation voltages, as opposed to using threshold voltages, in Supplementary Fig. S3. (**d**) Classification of extracellular AP traces and neuronal compartments. Orthodromic traces are classified as initial trace (It, marked in red), first orthodromic trace (1^st^Ot, in orange), subsequently appearing traces in the range of the proximal axon (Prox. Axon, in green), and later traces recorded at distal axonal regions (Dist. Axon, denoted in blue). Backpropagating traces are classified as first backpropagating trace (1^st^Bpt, in orange) and later traces in somatodendritic compartments (Sdt, in yellow). (**e**) Activation reliabilities (top) and activation failing points (bottom) from 4 neurons for 6 classes of stimulation electrodes are presented. Activation reliability represents the percentage of successfully evoked responses, whereas activation failing points represent the number of stimulations, after which activation reliability dropped below 95%. Neuronal morphology in (**a**–**d**) was obtained through lipofection.

We investigated the relationship between stimulation location and reliability of neuronal responses to high-frequency threshold stimulations. We found that electrodes recording AP’s initial trace provided the highest reliability, whereas stimulation sites towards distal axonal regions featured a gradually decreased fidelity of neuronal responses. High-frequency stimulation of somatodendritic compartments yielded the lowest reliability. Stimulation locations were selected based on spike-triggered average footprints (Fig. 8a). Traces were ranked according to their occurrence times and divided into orthodromic and backpropagating groups. The orthodromic group was further classified into (1) initial trace, (2) first orthodromic trace, (3) next-appearing traces conveyed by proximal axon, and (4) late-appearing traces recorded at distal axonal regions; the backpropagating group was classified into (5) first backpropagating trace and, (6) later appearing traces conveyed by somatodendritic compartments (see illustration in Fig. 8d). To verify that classified signals matched neuronal morphology, we superimposed spike-triggered average footprints and micrographs of lipofected neurons (Fig. 8a). We produced excitability profiles and defined stimulation thresholds for electrodes underlying the six classes of traces. Selected electrodes were next stimulated at threshold with 100 stimulation sequences, each comprising 100 biphasic pulses streamed at 100 Hz. Intervals between stimulation sequences were 10 seconds. The percentage of successfully evoked responses was expressed as the neuron’s activation reliability, whereas stimulation count at which activation reliability dropped below 95% was defined as the neuron’s activation failing point (Fig. 8c). Measures of activation reliability and activation failures were assigned to corresponding stimulation electrodes in order to construct a neuron’s activation reliability map (Fig. 8b). Activation reliabilities and failing points obtained from 4 neurons are jointly presented in Fig. 8e. Stimulation sites that required the lowest stimulation voltage to effectively activate the neuron were found to provide the most reliable neuronal response to high-frequency stimulation. To exclude the possibility that reliability of the neuronal response is an artifact of using different stimulation voltages (i.e., stimulation was applied at threshold voltages), we reproduced the activation reliability profiles of the two most reliable sites and of one highly unreliable site (presented in Fig. 8b,c) using the highest voltage found in the stimulation map (±400 mV). As expected, we found that suprathreshold voltages only improved reliability performances (Supplementary Fig. S4a,b).

## Discussion

We cultured neurons taken from whole primary neocortices over HD-MEAs and developed a set of recording and stimulation strategies to electrically visualize individual neurons in the network regardless of their activity level. Immunocytochemistry and live imaging were used to correlate extracellular electrical activity of different neuronal compartments with their morphology and verified our methods to electrically identify the AIS, axonal trunk and lower order branches. Extracellular traces enabled electrical detection of soma and proximal dendrites, however, unambiguous localization of these compartments required optical verification (Fig. 6a). Because of their small extracellular signals, distal dendrites were not detectable with our technology. As our intention was to develop methods for accessing any neuron in the network, we did not discriminate neuronal subtypes, which could have led to some of the variation in the results. However, in our experimental design we took steps to maximize the heterogeneity of the sample in order to obtain fairly generalizable results and minimize possible biasing (see Method section for details).

Consistent with previous observations, we found the AIS to produce the largest and first recorded extracellular APs, which enabled us to detect its location on electrical basis (Fig. 6a,d); previous data show the first recorded AP detected in the distal AIS in 44 out of 49 neurons, whereas the largest AP was recorded either near proximal AIS or near the peak of AnkG signal in 45 out of 49 neurons (D. Bakkum, 2016, manuscript submitted). In addition, positional variations of the AIS have been observed to affect the location of the largest AP trace. For example, in cells with the AIS being close to the soma, the largest AP trace typically also was found near the soma, whereas in cells with where the AIS was at larger distance from the soma, the largest AP trace also was shifted towards this more distant AIS position, whereas much smaller signal ampliudes were found near the soma. The soma and proximal dendrites provided minor contributions to the extracellular field, which, in many instances, were masked by the much larger AIS signals (D. Bakkum, 2016, manuscript submitted). With much smaller amplitudes, individual axonal traces were still detectable thanks to consistent extracellular waveforms across multiple stimulations^19^,^39^ that could be averaged together. The spatiotemporal distribution of AP footprint enabled to electrically visualize large portions of the axonal arbor and to track AP propagation in multiple axonal branches. The axonal tracing was possible by observing the relative changes in spatial position of the signal’s peaks across consecutive 50-μs time intervals (please see Supplementary Movie S1,2,3,4–5). While overall variations in AP amplitudes across different axonal locations were evident, this variability was not strongly dependent on the Euclidean or axial distance from the largest-amplitude trace of the footprint. We were able to record signals of axonal elements at large distance from the soma or AIS in many instances. This analysis was based on data obtained from 8 neurons from 8 different cultures. HD-MEAs and whole-cell patch-clamp were combined in order to correlate extracellular electrical features of axonal and somatodendritic compartments with their responsiveness to extracellular stimulation. In comparison to other neuronal regions, threshold stimulation at the AIS required the lowest voltage and provided immediate, selective and reliable orthodromic activation of the cell. Axonal stimulation revealed significant variations in the threshold voltages, which were discontinuously dispersed across the entire arbor. Threshold voltages of highly excitable axonal hotspots were comparable to that of the AIS and provided selective antidromic activation. Somatodendritic compartments either required high stimulation voltages to initiate an AP or did not initiate an AP for voltages within a physiologically harmless range^40^. When somatic stimulation was effective, evoked APs occurred after a delay, and reliability dropped dramatically for high frequency stimulation. Subthreshold somatic stimulation, however, depolarized the resting membrane potential without a delay, whereas subthreshold depolarization in the soma was not observed after axonal or AIS stimulation.

The presence of both high and low stimulation thresholds at proximal neuronal compartments (Figs 5b and 7a) resulted in lack of correlation between stimulation thresholds and individual parameters of extracellular APs (see Supplementary Fig. S2). Such variability might be explained by discontinuous distribution and abrupt differences in densities of voltage-gated ion channels in different neuronal elements^41^,^42^. This explanation is supported by the fact that the lowest thresholds are found near the location of the AIS (with high density of voltage-gated ion channels^41^; Fig. 6), while somatodendritic compartments (with lower density of voltage-gated ion channels^42^) require significantly higher voltages to trigger an AP (Fig. 7a) and perform unreliably during high-frequency stimulation (Fig. 8). While a multivariate combination of the extracellular AP features revealed a general correlation with stimulation thresholds (Fig. 6b), the obtained model did not enable to predict the most excitable sites, we expect for these reasons.

Owing to its small diameter, low local capacitance and high density of voltage-gated ion channels, the AIS is a highly effective integrator of postsynaptic charges and energetically favorable site for AP initiation^41^. These properties also give sensitivity to extracellular electric charges, rationalizing our finding that the AIS is the site with the lowest threshold for the extracellular stimulation. Supporting our results, sites with the lowest threshold for extracellular stimulation of retinal ganglion cells were found to reside nearby the soma^24^ and in the proximal axonal compartment enriched in voltage-gated sodium channels^25^. Similar results were provided by modeling studies^27^,^28^. The low stimulation thresholds allowed for selective neuronal activation. Increasing stimulation voltage influences a larger volume, eventually co-activating multiple neurons and losing selectivity. Indicatively, changing the stimulation site from the most sensitive to a neighboring electrode typically required an increase in the stimulation voltage to effectively activate the target neuron (see Figs 4a and 5b), which often resulted in loss off selectivity. This suggests that selective stimulation at the AIS is feasible through a very localized delivery of electrical charges, which can be achieved with low stimulation voltages and by having small electrode sizes. Supporting evidence from others show the effect of extracellular microstimulation is highly local^43^. While threshold stimulation of the AIS provided immediate neuronal activation, subthreshold stimulation at the AIS did not affect patch recordings at the somatic membrane (see Fig. 7).

High-frequency stimulation reliably evoked APs when applied at the AIS, possible due to Kv1 channels. By regulating AP half-width, Kv1 channels enable rapid recovery of sodium channels from inactivation, preventing AP failures in the AIS even for high-frequency neuronal activity^41^,^44^. In our data, activation reliability gradually decreased with distance from the AIS towards somatic and axonal locations (see Fig. 8). Interestingly, a similar trend in the distribution of Kv1 channel density was reported previously^41^, suggesting that reliability of neuronal responses to a high-frequency stimulation could be related to the distribution of Kv1 channels.

High stimulation thresholds along with delayed and highly unreliable neuronal responses of somatic extracellular stimulation are likely attributed to its large surface area and lower density of voltage-gated sodium channels compared to the AIS^42^. Supporting our findings, there is substantial evidence from experimental studies suggesting that electrical stimulation activates preferably axonal elements^19^,^21^,^23^,^24^,^25^,^26^. For example, early experimental studies reported that thresholds for the activation^23^ of spinal motorneurons and spinal border cells was lowest near the AIS, more than two-times higher near the soma, and up to five times higher at dendritic stimulation positions^23^. Studies performed in cortical gray matter suggest that axons, but not cell bodies, are neuronal elements activated by extracellular stimulation^21^. Because experimental studies have been limited by techniques and methods available at times, modeling studies have been providing important insights into biophysical aspects of extracellular stimulation^45^, leading to better understanding about which neuronal elements are activated by electrical charges^20^,^46^,^47^.

In extracellular electrophysiology, antidromic stimulation is a common method to evoke neuronal activity. Except for ‘ectopic’ APs that have been reported to initiate in distal axons^48^,^49^,^50^, antidromic stimulation does not replicate the direction of propagation of spontaneous axonal APs. Extensively branched axons overlap with other neurons and form a net of excitable spots. This typically prevented us from achieving selective activation of individual neurons at sites in the axonal arbor. Moreover, simultaneous activation of multiple branches might lead to annihilation of propagating APs, producing patterns of AP propagation nonexistent or rare under normal physiological conditions^49^.

We found that axonal sensitivity to extracellular stimulation broadly varied across an axonal arbor, whereas highly reactive hotspots revealed stimulation thresholds approaching that of the AIS (Fig. 4a). Stimulation efficacy is dependent on the relative proximity of neurites to an electrode, which likely varies across axonal arbors. Thus for example, axonal regions that are tightly attached to the electrode surface could give highly reactive hotspots, whereas regions that are between electrodes could give low-reactive spots. In addition, the presence of glial cells may locally affect conductance near individual electrodes, may elevate axons away from the array’s surface making them less accessible to stimulation, or, on the other hand, might cover an axon over an electrode, thereby reducing spreading resistance and increasing stimulation effectiveness. Highly reactive axonal hotspots did provide selective antidromic activation in many cases (Supplementary Movie S1,3).

The presented method to selectively stimulate, and at the same time, to non-invasively record signals in multiple axonal branches of a single neuron, circumvents technical limitations that accompany electrophysiological and optical techniques commonly used in axonal recordings, and as such, allows for studying new aspects of information processing in mammalian axons^51^ over extended periods of time. Reliable stimulation at high-frequencies, for example, allows for studying modulation of axonal APs^52^,^53^ or mechanisms of axonal conduction failures^54^,^55^. Non-invasive control over a soma’s subthreshold potential is useful for studying spatiotemporal aspects of analog-digital integration of axonal signals^56^,^57^. Flexible stimulation sites enable reproducing and studying ectopic APs, as well as annihilation of axonal signals^48^,^49^. Furthermore, precise control over the spike timing of individual neurons enables manipulating synaptic plasticity, and thereby, reorganizing network connectivity^58^. This is useful, for example, to study the role of spike-timing dependent plasticity in organizing neuronal assemblies^2^ and to manipulate network dynamics in closed-loop learning tasks^59^,^60^. Finally, knowledge arising from basic *in vitro* studies can help inform the design of *in vivo* applications^25^. For example, understanding how individual neurons respond to electrical stimulation on a subcellular level, and how selective stimulation can be achieved, can aid the design of prosthetic implants and brain-machine interfaces.

## Methods

### Animal use

All experimental protocols were approved by the Basel Stadt veterinary office according to Swiss federal laws on animal welfare and were carried out in accordance with the approved guidelines.

### HD-MEA

A complementary-metal-oxide-semiconductor (CMOS) -based HD-MEA system, fabricated in a 0.6-μm CMOS 3M2P process was used for extracellular neuronal recording and stimulation^13^. The electrode array has been co-integrated with circuitry units on the same chip and features a total of 11,011 electrodes (active surface of 8.2 × 5.8 μm^2^ per electrode) within an area of 1.99 × 1.75 mm^2^, providing a density of 3,150 electrodes per mm^2^ (17.8-μm center-to-center pitch). The chip surface has been passivated with a stack of alternating SiO_2_ and Si_3_N_4_ layers. Bond-wires are encapsulated in epoxy (Epo-Tek 302–3M, John P. Kummer AG, Cham, Switzerland). Owing to a flexible switch matrix and 13,000 static random-access memory cells integrated underneath the array, up to 126 readout and/or stimulation channels can be routed to the desired electrodes and reconfigured within a few milliseconds. Electrodes exhibit signal-to-noise ratios up to 180σ_noise_ and between 1 and 20σ_noise_ for somatic and axonal signals, respectively. On-chip circuitry is used to amplify (0–80 dB programmable gain), filter (high pass: 0.3–100 Hz, low pass: 3.5–14 kHz), and digitize (8 bit, 20 kHz) neuronal signals. Digitized signals are sent to a field-programmable gate array (FPGA) board and further streamed to a host PC for real-time visualization and data storage. Recorded signals were up-sampled to 200 kHz following the Whitaker–Shannon interpolation formula. Matlab R2012a was used for data analysis and to design extracellular stimulation protocols.

### Platinum-black deposition

We deposited platinum black on the electrodes in order to reduce their impedance and to increase the effective electrode-neuron interfacing area. Consequently, stimulation voltages needed to elicit neuronal responses were lowered 3–5 times, which significantly improved stimulation performance of the array. A 180 mA current was simultaneously applied to all electrodes for 45–75 s while using a platinum counter electrode immersed in the deposition solution (0.7 mM hexachloroplatinic acid and 0.3 mM lead (II) acetate anhydrous). Deposition uniformity was verified optically under a microscope and electrically by measuring the impedance of electrodes (see below).

### Impedance measurement

The consistency of experiments in this study required equal stimulation performances of all electrodes. To verify that all electrodes provide even current flow, we measured impedance in response to a test voltage signal applied at each electrode. We found negligible variations in impedance (<1%) across all electrodes in 8 chips. All measurements were performed in phosphate-buffered saline (PBS; Sigma, Buchs, Switzerland) by using an external DS360 ultra-low distortion function generator (Stanford Research Systems) to send voltage pulses and the integrated amplifiers on the HD-MEA system for readout. The external function generator sent a sinusoidal voltage signal (V_stim_; 1mV_pp_ at 1 kHz) through the chip’s reference electrode into the PBS medium, whereas active electrodes were used to record the resulting signal through on-chip readout channels. The attenuation of the recorded signal is dependent on the impedance of the active electrode (Z_e_) and that of the amplifier input (Z_in_). The amplifier inputs of acquired signals were I-Q demodulated in Matlab R2012a in order to extract the amplitude of the input reference signal (V_meas_) at 1 kHz.

### Cortical cultures

We used a culturing protocol developed for long-term maintenance of neural cultures^61^. We introduced minor adaptations to the protocol in order to constrain culture growth to the area of the array and to maintain optimal conditions of growth media during long-term experimentation. Cortices from embryonic day 18 Wistar rat were dissociated enzymatically in trypsin with 0.25% EDTA (Life Technologies, Bleiswijk, Netherlands) and physically by trituration. For cell adhesion, a layer of 0.05% polyethyleneimine (Sigma) in borate buffer (Chemie Brunschwig), followed by a layer of 0.02 mg ml^−1^ laminin (Sigma) in Neurobasal (Life Technologies) was deposited on the electrode array. To constrain culture growth to the electrode array, a cell-drop covering ~3 mm^2^ was seeded in the center of the array. We plated 1,000–10,000 and 20,000–40,000 cells to grow low- and high-density cultures. The plating media were changed to growth media after 6 days (for low-density cultures) or 24 h (for high-density cultures). Plating media consisted of Neurobasal supplemented with 10% horse serum (HyClone), 0.5 mM GlutaMAX and 2% B27 (Life Technologies). Growth media consisted of DMEM (Life Technologies) supplemented with 10% horse serum, 0.5mM GlutaMAX and 1mM sodium pyruvate (Life Technologies). Cultures were maintained inside an incubator with controlled environmental conditions (36 °C and 5% CO2). Experiments were conducted at 14–28 DIV. The culturing chambers were sealed with a ~1 mm layer of light mineral oil (Sigma) floating above the growth medium. The sealing provided selective permeability to gasses, such as O_2_ and CO_2_, and prevented evaporation and consequent changes in growth media’s osmolarity during long-term experiments. To block synaptic inputs, we inhibited GABA-R, NMDA-R and AMPA-R by bath application of 50 μM bicuculline methiodide, 100 mM 2-amino-5-phosphonovaleric acid and 10 mM 6-cyano-7-nitroquinoxaline-2, 3-dione (CNQX; Sigma), dissolved in growth media.

### Live imaging

Live-cell visualization of whole neurons was performed either by transfection or by calcium imaging. Transfection was performed using pLV-hSyn-RFP plasmid from Edward Callaway (Addgene plasmid # 22909) and Lipofectamine 2000 (Life Technologies) in accordance with the manufacturer’s protocol. Transfections were carried out in serum-reduced OptiMEM media (Life Technologies). Calcium imaging was performed using Fluo-4 AM (1mM solution; Life Technologies F-14217) and Pluronic F-127 (20% Solution in DMSO; Life Technologies P-3000MP) in accordance with the manufacturer’s protocol. Calcium imaging was carried out in HBSS (Life Technologies).

### Sample representativity

We studied in total 52 different neurons in 27 preparations. We took several measures to obtain representative and generalized results. First, all experiments were performed on developed cortical cultures (at 14–28 DIV), where all neuronal subtypes are expected to be mature and electrically identifiable^62^. Second, the sampling included both, neurons whose axonal arbors covered large areas, and neurons whose axons occupied comparably small areas in the culture. Third, the sampling included spontaneously active and silent neurons. Fourth, the group of spontaneously active neurons included cells exhibiting different firing frequencies. For example, we sampled occasionally spiking neurons (<0.5 Hz), tonically active neurons (~0.5–~1 Hz) and bursting neurons (>1 Hz).

### Network activity mapping

Cortical neuronal cultures are spontaneously active, and a sequential scan of 95 recording configurations captures activity in the entire array. In each recording configuration, up to 126 randomly selected electrodes sampled neuronal activity for a duration of 60 seconds. By randomizing the recording sites, the activities of same neurons in the network could be sampled across multiple recording configurations. The average voltage traces, recorded by each electrode, were extracted and used to reconstruct the map of the network’s electrical activity (Fig. 1a).

### Spontaneous AP footprints

Simultaneous access to signals from proximal compartments of the neuron enabled us to reconstruct the spatial distribution of averaged extracellular AP waveforms, referred to as the spontaneous AP ‘footprint’ (Fig. 1b). To obtain these signals, we used high-density recording configurations to scan neuronal locations pre-chosen from the activity maps (Fig. 1a). In each configuration, blocks of 6 × 18 electrodes were connected to read-out channels to sample neuronal activity for 5 minutes. Depending on the experimental aims, we used 2 up to 8 partially overlapping recording blocks in order to sample signals conveyed by a single or by multiple neurons. Extracellular signals arising from different neurons were sorted by using the UltraMegaSort2000 software^63^, and spontaneous AP footprints were reconstructed by using custom-designed Matlab code.

### Spike-triggered averaging

Array-wide averaging of voltage traces, synchronized with the earliest emerging trace (initial spike) found in the neuron’s spontaneous AP footprint, enabled us to reconstruct the spatiotemporal distribution of extracellular AP waveforms arising from axonal arbors (Fig. 1c,d). The first step in obtaining these data was selecting electrodes with the 4 largest spike amplitudes in the spontaneous AP footprint. We next designed multiple recording configurations covering the entire array, where, in each configuration, 4 of the 126 read-out channels were set as the 4 preselected electrodes. Other available channels were connected to randomly selected electrodes. Each configuration was used to sample neuronal activity during 2 minutes. Spikes recorded by the 4 preselected electrodes in each configuration were sorted by using the UltraMegaSort2000 software^63^, and the timestamps of the neuron’s initial spike were extracted. Array-wide spike-triggered average signals were computed, and the footprint was reconstructed by using custom designed Matlab code.

### Stimulation-triggered recordings

We used balanced positive-first biphasic voltage pulses because of their effectiveness in electrical stimulation^38^. Voltage pulses had phase durations of 200 μs and amplitudes between ±10 and ±1000 mV. Equivalent charge values for our voltage stimulation can be approximated as follows: *q* = *VC*; where *q* represents the charge in picocoulombs (pC), *V* stands for the voltage in millivolts (mV), and *C* represents the capacitance in nanofarads (nF), the latter of which amounted to approximately 1.6 nF for the MEA electrodes (with surface of 8.2 × 5.8 μm^2^; also see ref. 18). Stimuli were applied to one electrode at a time in all stimulation protocols. Stimuli were applied at a frequency of 4 Hz (unless specified differently in the Result section). This particular frequency was chosen because it enabled us to limit the duration of our experiments (to max 2 hours) and, at the same time, to minimize possible effect on intrinsic neuronal plasticity, which could be induced by long-lasting stimulations at higher frequencies^34^. In our experimental design, we used different stimulation-triggered recording strategies. To detect the array-wide responses to an electrical stimulation, we scanned the array with 95 randomly organized configurations covering the entire array, whereas stimuli were applied at the same single electrode multiple times per configuration. Stimulus-triggered responses recorded throughout each configuration were temporally aligned with regard to the downswing of the biphasic stimulation. Aligned responses were used to reconstruct the footprints (Fig. 2) and make supplementary movies (Supplementary Movie S2 and 5). To detect stimulus-triggered responses of a single neuron, we designed a custom configuration with up to 125 electrodes underlying multiple compartments of the neuron. One electrode was connected to a stimulation channel. In cases where the experimental design required sequential stimulation across the entire neuron, many complementary configurations were designed. Each configuration comprised a single stimulation site and a maximal number of readout electrodes underlying detectable neuronal compartments (Fig. 3b).

### Movies and neuronal contours

Stimulation- and spike-triggered average neuronal signals captured across the entire array were used to represent spatiotemporal distributions of extracellular APs in a movie format (see Supplementary Movies S1–5). Observing the spatial movement of signal peaks in consecutive movie frames enabled us to visually track AP propagation and to manually produce neuronal contours as presented in Figs 1, 2, 3, 4. In these figures, the axonal contours are only meant to serve as a guide to the eye in order to better visualize the spatiotemporal distribution of the corresponding AP footprint.

### Combined HD-MEA and patch clamp recordings

The experimental setup includes the HD-MEA system combined with an upright microscope (Leica DM6000B) and a conventional patch clamp system (Multiclamp 200B amplifier, Sutter Instruments micromanipulator). The microscope is mounted on a motorized XY-stage (Scientifica UMS) allowing for imaging of a large working area and storage of the precise microscope position for every acquired image. Custom image alignment software written in Matlab was developed to automatically align the acquired image with the corresponding HD-MEA coordinates. An FPGA based setup was extended with four analog-to-digital conversion channels (ADCs, AD974 Analog Devices) on a custom printed-circuit board for synchronous acquisition of the patch clamp signals together with the HD-MEA data. For the patch clamp experiments, the growth medium was replaced with a HEPES-buffered external bath solution (149 mM NaCl, 3.25 mM KCl, 2 mM CaCl_2_, 2 mM MgCl_2_, 10 mM HEPES and 11 mM Glucose) adjusted to pH 7.35. The bath was constantly perfused during the experiment, and all experiments were performed at room temperature. Neurons grown on top of the array were visualized in bright-field illumination using differential interference contrast optics on the upright microscope. The patch clamp micropipettes (borosilicate glass, Sutter Instruments) had resistances of 5–7 MΩ and were filled with an internal solution (135 mM C_6_H_11_KO_7_, 20 mM KCL, 2 mM MgCl_2_.6H_2_O, 10 mM HEPES, 0.1 mM EGTA, 2 mM Na_2_ATP and 0.3 mM Na_3_GTP) adjusted to a pH of 7.3. Alexa Fluor 594 (Life Technologies) was added to the internal solution, and fluorescence images were acquired during and after the patch clamp experiment. The patch clamp amplifier was controlled using the open-source software WinWCP (John Dempster, University of Strathclyde, UK).

### Immunocytochemistry

Cortical cultures were fixed in 4% paraformaldehyde (Life Technologies) in PBS (Sigma) at pH 7.4 for 15 min at room temperature, washed twice with ice-cold PBS, permeabilized with 0.25% Triton X-100 (Sigma) in PBS for 10 min, and washed three times in PBS. Fixed cultures were exposed to phosphate-buffered saline with tween 20 (1% bovine serum albumin and 0.1% tween 20 in PBS; Sigma) for 30 min to prevent unspecific binding of antibodies. The primary antibodies Anti-MAP2 (Abcam) and Anti-Ankyrin G (Abcam), diluted in phosphate buffered saline with tween 20 to a ratio of 1:500 and 1:200 respectively, were added and left overnight at 4 °C on a shaker. Cultures were washed three times in PBS for 5 min each time on the shaker. The secondary antibodies Alexa Fluor 647 (Life Technologies) and Alexa Fluor 488 (Life Technologies), diluted to ratio of 1:200 in PBS with 1% BSA, were added and left for 1 h in the dark at room temperature. Samples were washed three times in PBS for 5 min each time in the dark and then stored at 4 °C.

### Microscopy and image representation

A Leica DM6000 FS microscope, Leica DFC 345 FX camera, and the Leica Application Suite software were used to produce micrographs. For the sake of visual clarity, micrographs of neurons presented in Figs 5, 7 and 8 are displayed as cartoons produced by using Adobe Photoshop CS5 and Adobe Illustrator CS5.

## Additional Information

**How to cite this article**: Radivojevic, M. *et al*. Electrical Identification and Selective Microstimulation of Neuronal Compartments Based on Features of Extracellular Action Potentials. *Sci. Rep.* **6**, 31332; doi: 10.1038/srep31332 (2016).

## Supplementary Material

Supplementary Movie 1

Supplementary Movie 2

Supplementary Movie 3

Supplementary Movie 4

Supplementary Movie 5

Supplementary Information
