# Supplementary material for: Electrical Identification and Selective Microstimulation of Neuronal Compartments Based on Features of Extracellular Action Potentials
Source: Sci Rep. 2016 Aug 11;6:31332. doi: 10.1038/srep31332 (PMC4980679; doi:10.1038/srep31332)

Electrical Identification and Selective Microstimulation of Neuronal Compartments Based on Features of Extracellular Action Potentials

Milos Radivojevic*, David Jäckel, Michael Altermatt, Jan Müller, Vijay Viswam, Andreas Hierlemann and Douglas Bakkum

ETH Zurich, Department of Biosystems Science and Engineering, 4058 Basel, Switzerland

milos.radivojevic@bsse.ethz.ch

SUPPLEMENTARY FIGURE LEGENDS

Supplementary Figure S1. Reliable detection of individual axonal APs triggered by extracellular stimulation

Exemplary waveforms of averaged and individual APs triggered consecutively from 4 different stimulation sites and recorded at 3 distal axonal compartments are presented in panels (a) to (d). Colors of averaged AP waveforms indicate occurrence times of negative AP peaks relative to the stimulation time. Individual APs are colored in gray. Gray axonal contours serve as guide to the eye and are estimated by observing relative changes in spatial position of the extracellular signal peaks across consecutive 50-μs time intervals, frame by frame. The four complete stimulation-triggered footprints are presented in Fig. 2a-d and in Supplementary Movie S2. The stimulation spots in Supplementary Fig. S1a-d correspond to those in Fig. 2a-d.

Supplementary Figure S2. Correlations between features of the spontaneous AP footprint and site-specific stimulation thresholds of proximal neuronal compartments

(a) Correlations between stimulation threshold values and individual parameters extracted from spontaneous AP footprints are presented in four graphs. Occurrence time of the negative peak in the traces (1); occurrence time of the minimum in the 1st derivative of the traces (2); negative peak amplitude of the extracellular traces (3) and peak-to-peak amplitudes (4) are correlated with the corresponding stimulation threshold values extracted from stimulation maps of 14 neurons. Most sensitive, second and third sensitive sites (Mss, 2^{nd}ss and 3^{rd}ss), are color-coded by red, orange and yellow respectively.

(b) Correlations between stimulation threshold values and individual parameters extracted from spontaneous AP footprints are presented in four graphs. Spatial distance of the stimulation site from the most sensitive site (1); distance of the stimulation site from the electrode featuring the peak trace (2); distance of the stimulation site from the footprint's center of mass (3) and distance of the stimulation site from the location of the initial trace (4) are correlated against corresponding stimulation threshold values extracted from stimulation maps of 14 neurons. Most sensitive, second and third sensitive sites (Mss, 2^{nd}ss and 3^{rd}ss), are color-coded by red, orange and yellow respectively.

Supplementary Figure S3. Spatiotemporal distribution of extracellular AP waveforms with regard to neuronal morphology

Spike-triggered footprint superimposed over a micrograph of the corresponding lipofected neuron. Colors of AP waveforms indicate timing of their occurrence relative to the first recorded activity. In the insets magnifying two axonal portions the distribution of axonal AP waveforms is displayed. The same neuron is presented in Fig. 5, 8, Supplementary Fig. S4, and Supplementary Movie S5.

Supplementary Figure S4. Influence of suprathreshold stimulation voltages on neuron's activation reliability

(a) (Left) Activation reliability map of threshold stimulation. Circle sizes indicate relative activation reliability (%), and the colors represent activation failing points (no triggering of an AP, see Fig. 8). Activation reliability represents the percentage of successfully evoked responses, whereas the activation failing point represents the number of stimulations after which activation reliability dropped below 95%. Three electrodes (numbered as 3, 4 and 8) were stimulated at 100 Hz at threshold voltages. (Right) Reliability profiles for electrodes 3, 4 and 8 in the activation reliability map. Red dashed lines indicate the activation failing thresholds and red circles projected on the x-axes indicate activation failing points.

(b) (Left) Activation reliability map of suprathreshold stimulation. Circle sizes indicate relative activation reliability (%), and the colors represent activation failing points (no triggering of an AP, see Fig. 8). Activation reliability represents the percentage of successfully evoked responses, whereas the activation failing point represents the number of stimulations after which activation reliability dropped below 95%. Three electrodes (numbered as 3, 4 and 8) were stimulated at 100 Hz at suprathreshold voltages. (Right) Reliability profiles for electrodes 3, 4 and 8 in the activation reliability map. Red dashed lines indicate activation failing thresholds and red circles projected on the x-axes indicate activation failing points.

Supplementary Figure S1

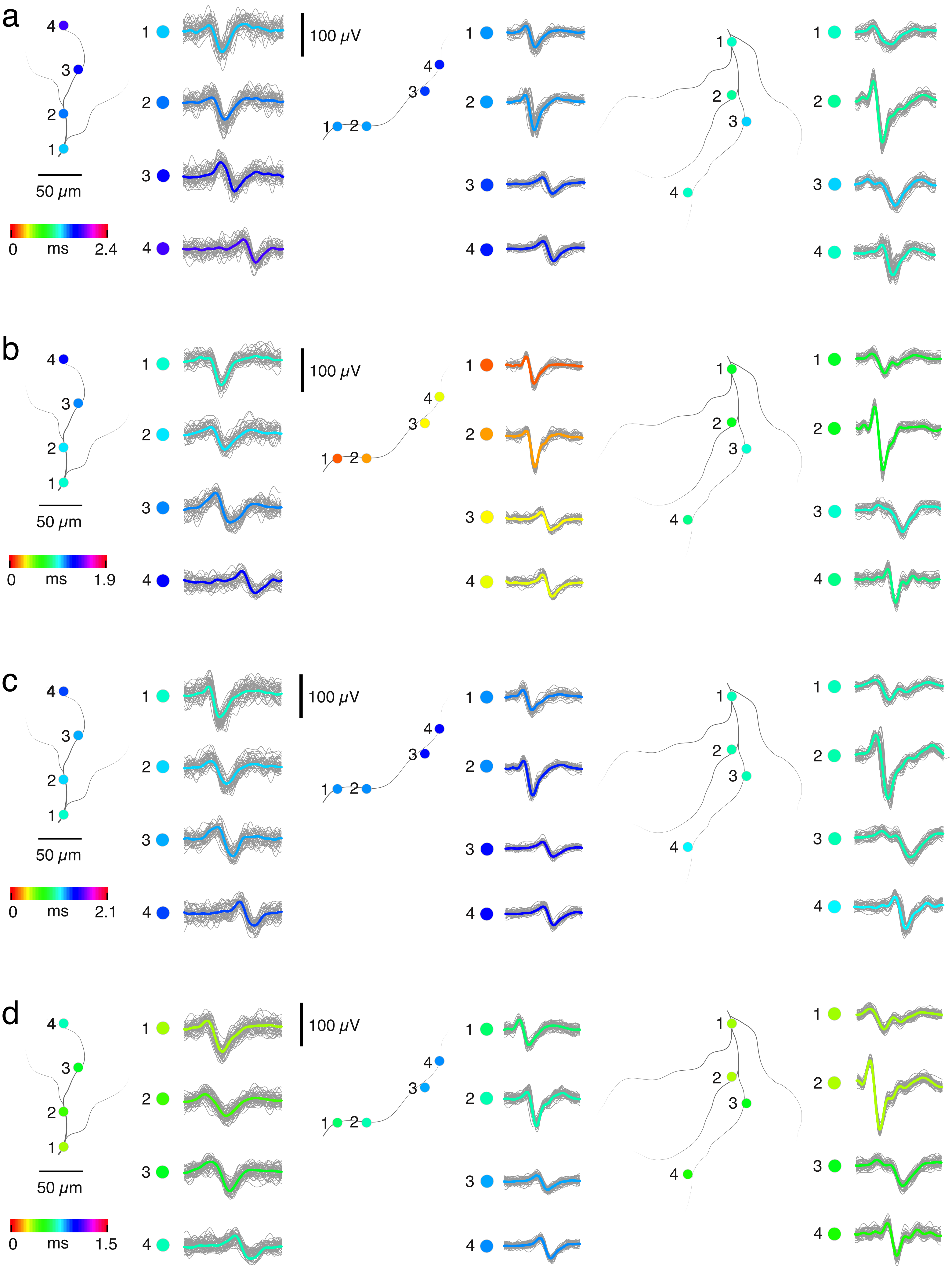

Supplementary Figure S2

a

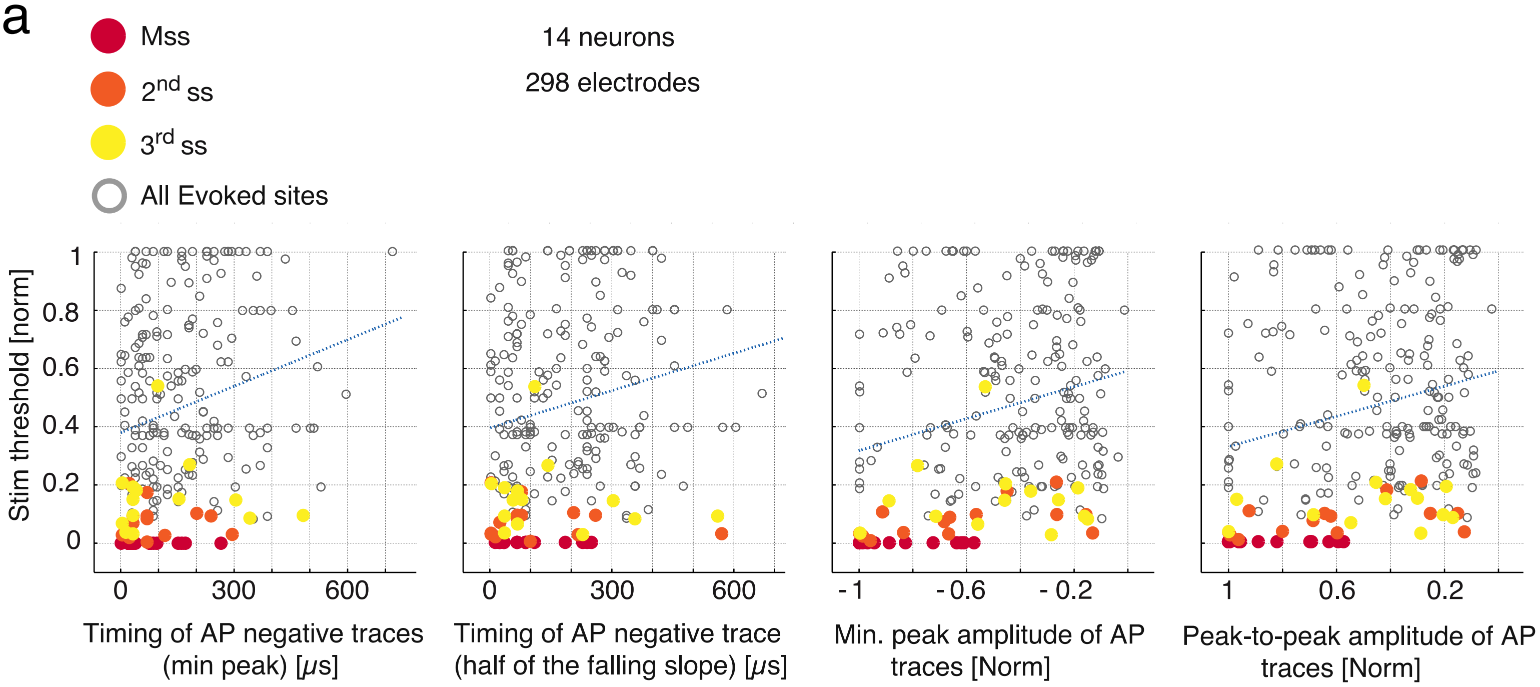

b

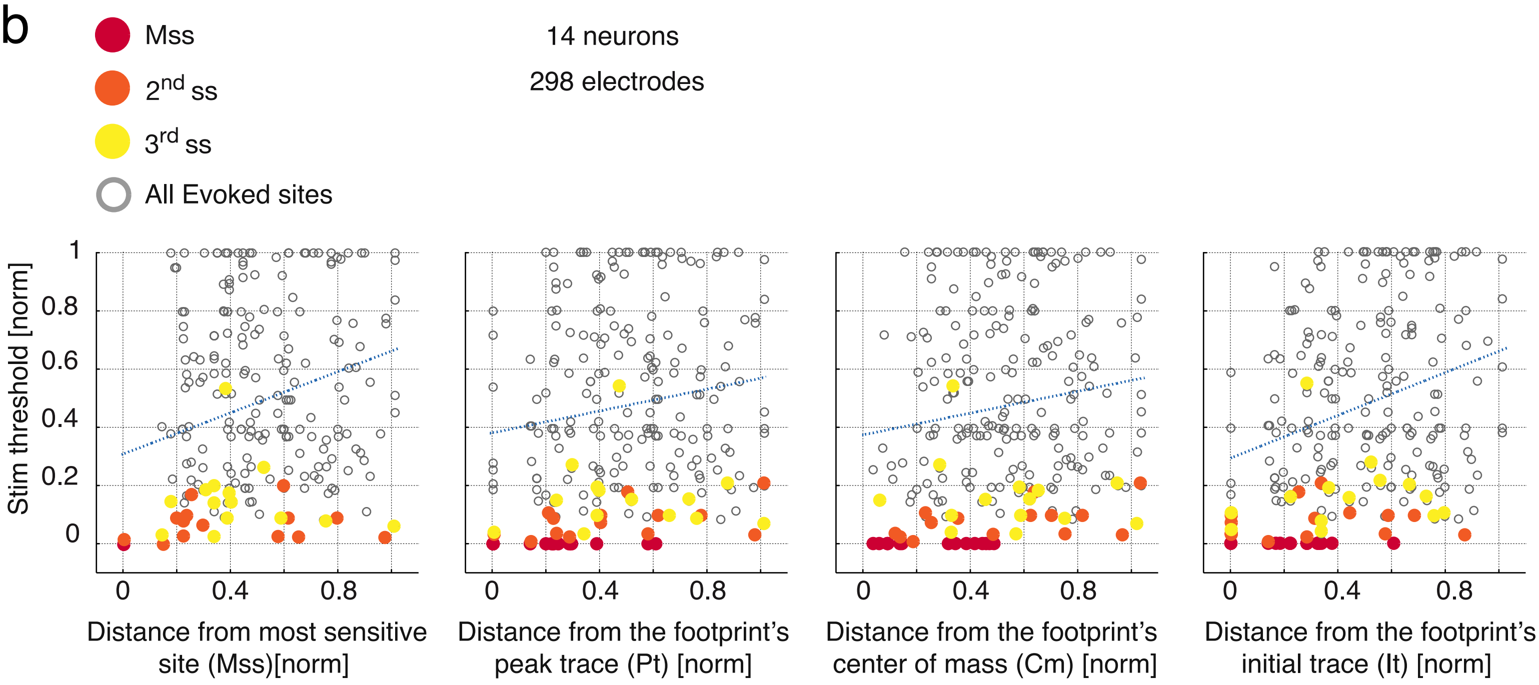

Supplementary Figure S3

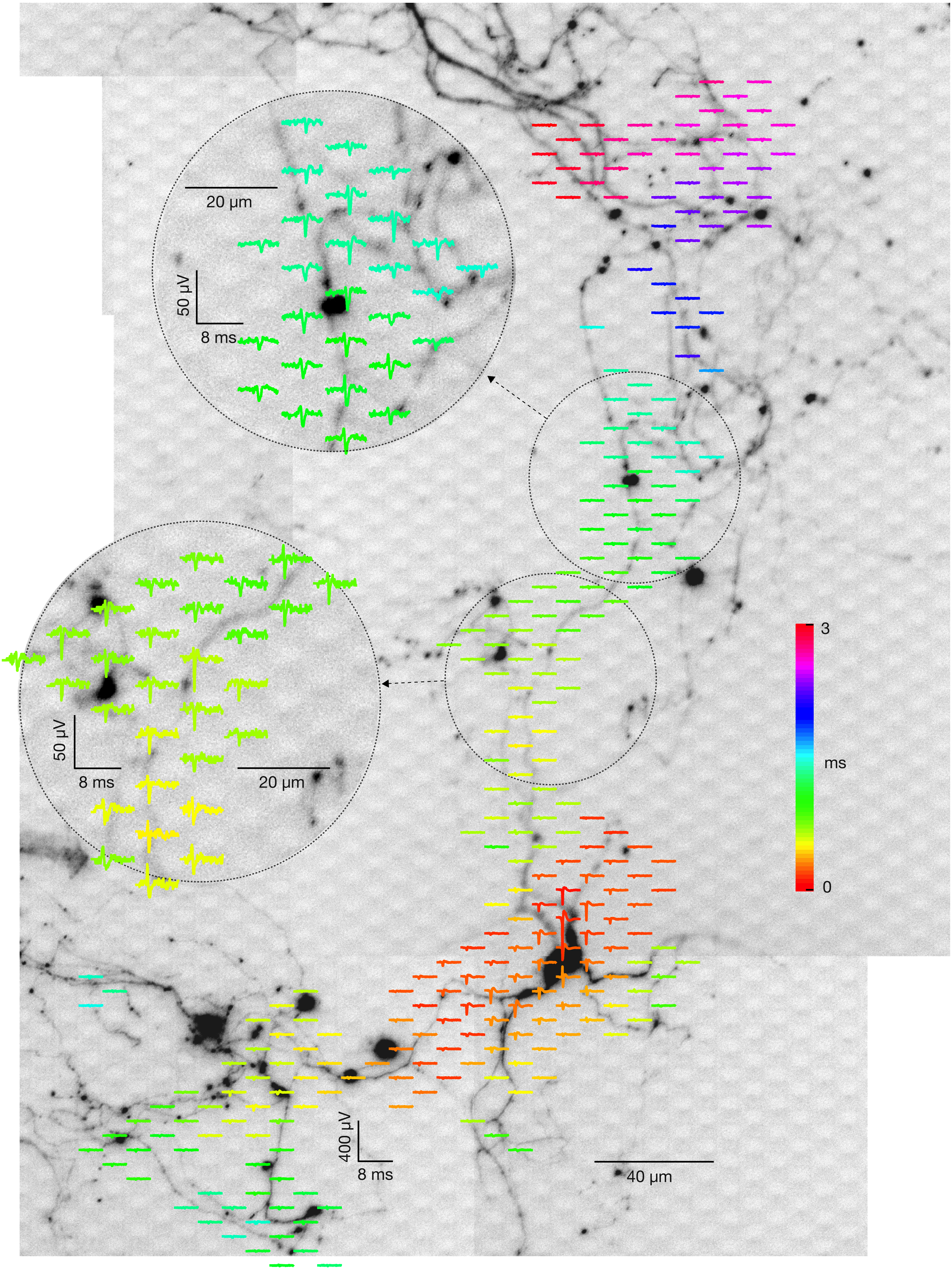

Supplementary Figure S4

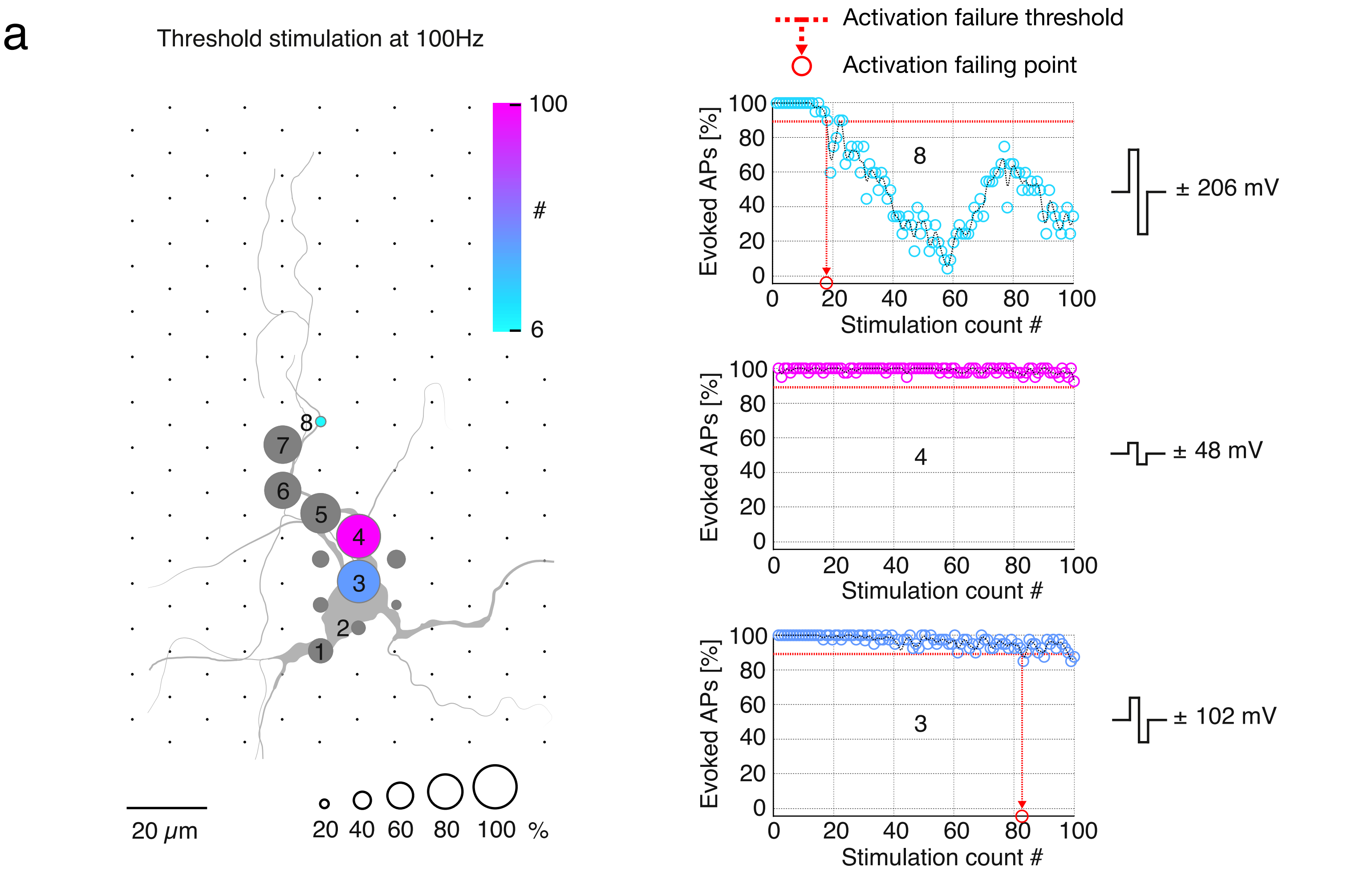

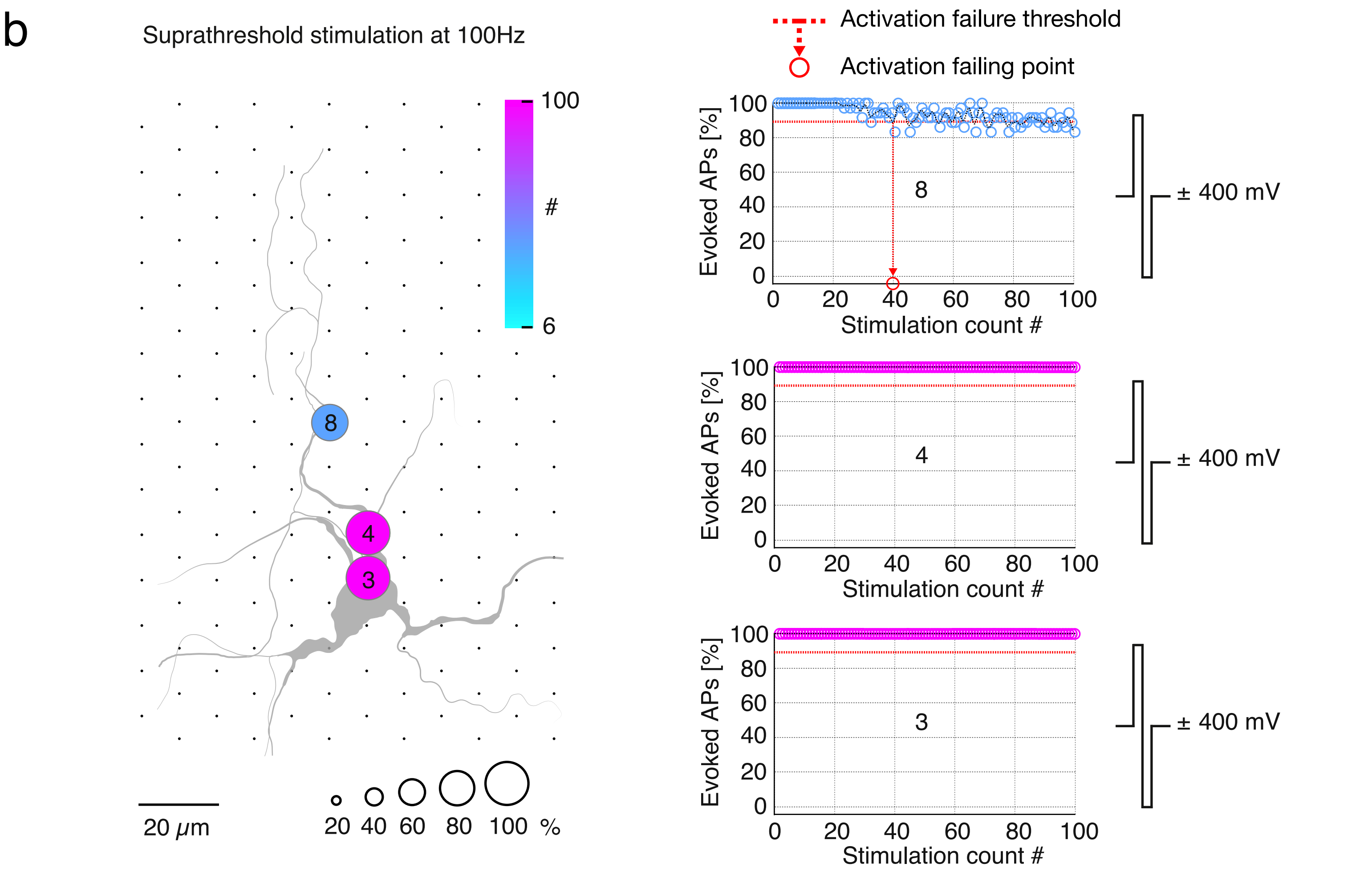

Supplement: Supplementary Information [file srep31332-s6.pdf]
